# Strengths and weaknesses of the South-South Learning Exchange: a qualitative analysis of experts’ perspectives

**DOI:** 10.12688/gatesopenres.14699.1

**Published:** 2023-08-18

**Authors:** Isotta Triulzi, Rita Kabra, Komal Preet Allagh, James Kiarie

**Affiliations:** 1 Institute of Management, Scuola Superiore Sant'Anna, Pisa, 56127, Italy; 2 Department of Sexual and Reproductive Health and Research, World Health Organization, Geneva, 1211, Switzerland; 3 Consultant, Department of Sexual and Reproductive Health and Research, World Health Organization, Geneva, 1211, Switzerland

**Keywords:** learning exchange; South-South learning exchange; peer-to-peer; South-South cooperation; knowledge exchange

## Abstract

**Background:**

South-South learning exchange (SSLE) refers to an interactive learning process where peers exchange knowledge and experience to work towards a beneficial change. Despite organizations having recently increased the opportunity to run SSLEs, the SSLE support mechanisms and processes are not well documented in the scientific literature. This study explored experts’ perspective on SSLEs, strengths, weaknesses and mechanisms leading to outcomes.

**Methods:**

We conducted a qualitative study using semi-structured interviews on experience of participants and organizers of SSLEs. Data were collected between 1 ^st^ September 2021 to 26 ^th^ November 2021. All data were digitally recorded, transcribed verbatim, and analysed. In the analysis, we adopted an inductive approach derived from thematic analysis.

**Results**:

Sixteen experts who have participated in or facilitated one or more SSLE were interviewed. Experts’ accounts demonstrated an appreciation of participants’ empowerment, positive peer-to-peer “mind change” and convincing and powerful hands-on learning of this approach as strengths in the successful implementation of the SSLE. Being resource heavy, participant reluctance and absence of a validated methodology emerged as main weaknesses of the South-South learning approach, which could impair the effectiveness of this scheme.

**Conclusions:**

The SSLE is a promising approach to exchange knowledge and experience to work toward a desired change. This study suggested that this approach could gain robustness and credibility adopting a validated and systematic methodology. Furthermore, national and international funds improve availability of and accessibility to learning on the SSLE.

## Introduction

The South-South learning exchange or peer-peer learning exchange refers to an interactive learning process where peers exchange knowledge and experience to work towards a beneficial change ( WHO, 2018a). These exchanges are often referred as south-to-south cooperation or knowledge exchange. The WHO’s thirteenth “General Programme of Work (2019 -2023)” ( World Health Organization, 2018b) integrated south-to-south cooperation as a strategy to develop and scale up innovative solutions for building capacities through shared learning and equitable partnerships.

The exchanges allow participants to “learn firsthand from the experience of their peers how a challenge was solved or solution implemented” ( Kumar & Watkins, 2017) and they require coordination with two peer teams (knowledge seeker and knowledge provider), a facilitator (broker) who brings the two teams together, and various stakeholders that support the work towards a change ( World Health Organization, 2018b). SSLE can inspire peers, create new ideas, implement reforms, share practical problem-solving ‘how-to’ knowledge, and foster collaboration and advocacy ( The World Bank, 2019a) – all key factors for social learning processes ( Reed *et al.*, 2010). SSLE may be organized by international agencies, non-governmental organizations (NGOs) and governments. Different ways ( Mier-Alpano *et al.*, 2022) such as platforms have emerged to exchange knowledge over the years ( Food and Agriculture Organization of the United Nations (FAO), 2021) and organizations have increased the opportunity to run SSLE programs to share practices and experiences in various areas, such as development, climate change, conservation management, and reproductive health and rights ( Partners In Population And Development, 2021; The World Bank, 2019a). However, few guidelines are available for conducting an exchange and are focused on specific topics like fishery learning exchanges ( Thompson *et al.*, 2017). In addition, it is unclear if these guidelines arose from an “empirically-grounded, peer-reviewed process” ( Kumar & Watkins, 2017).

The WHO embarked on the Family Planning (FP) Accelerator project in 2019 ( World Health Organization, 2020) with the objective to improve access to quality and rights-based FP services. Under this project, ten low-income countries have participated in an SSLE. These exchanges follow a five-step approach using the preliminary version of “A step-by-step Guide to South–South learning exchanges” guide ( World Health Organization, 2018a), which allows to plan, conduct, and evaluate a SSLE. The steps include defining the need for and purpose of the learning exchange, planning the SSLE, facilitating the learning exchange, supporting implementation of the action plan and following-up after the learning exchange. An example is the SSLE between the Nepalese and Sri Lankan ministries of health, in which respective teams shared their best practices and learnings resulting in implementation of a web-based system for logistics management of FP commodities in Sri Lanka and Nepal started implementing integrated family planning services in a decentralized environment, using a lifecycle approach to improve the uptake of postpartum FP ( Kabra *et al.*, 2022).

The peer-reviewed literature reporting SSLE experiences is limited to specific topics such as conservation management ( Bretos *et al.*, 2017; Gardner *et al.*, 2017; Heyman & Stronza, 2011), information system in health ( Were *et al.*, 2019), human capacity on HIV/AIDS services ( Ivers *et al.*, 2010), disease outbreak and health system strengthening ( Olu *et al.*, 2017). Beyond scientific literature, results of previous SSLEs are illustrated in reports developed by organizations, such as the World Bank ( The World Bank, 2019b), the United Nations Development Programme ( United Nations Development Programme, 2017), the United Nations Office for South-South Cooperation ( United Nations Office for South-South Cooperation, 2019; United Nations Office for South-South Cooperation, 2020), and the United nations population fund ( United Nations Population Fund (UNFPA), 2018; 2021). Given the scarcity and thus great need for scientific publications on this topic Allagh *et al.*, 2023, the intent of this research was to understand the value of SSLEs. This paper explored experts’ perspective on SSLEs, strengths, weaknesses and mechanisms leading to outcomes.

## Methods

### Design

We conducted a qualitative study using key informant interviews to explore the experience of participants and organizers of SSLEs. We adopted an inductive approach derived from thematic analysis. This qualitative study follows the “Consolidated Criteria for Reporting Qualitative Research COREQ” ( Tong *et al.*, 2007) (Extended data).

### Research setting

This qualitative study is embedded into the WHO Family Planning Accelerator project, in which countries adopted a South-South learning approach. The project is overseen by the Contraceptive Unit at the Department of Sexual Reproductive Health and Rights at the WHO. The authors of this study have recently conducted a scoping review on the purposes, approaches, barriers, facilitators and outcomes of SSLE in FP ( Allagh *et al.*, 2023), which follows the six-step methodological framework suggested by Levac *et al.* ( Arksey & O’Malley, 2005; Levac *et al.*, 2010). The last step of this framework includes a stakeholder consultation that was preceded by key informant interviews with stakeholders. In this paper, we present a detailed analysis of the findings generated by these interviewees. These findings were the basis for the stakeholder consultation held at the WHO in November 2021, where stakeholders shared their experiences, enablers, barriers and lessons learnt with the goal of making future SSLEs more efficient and effective.

### Interviews

We conducted semi-structured interviews using a pre-structured interview guide (Extended file- interview guide), with open-ended questions that covered the following areas:

I. Process and methodology adopted during the SSLEs;
II. Participants’ personal and professional experiences on SSLE programs, including challenges, enablers, and lessons learnt.

We sampled purposively by reaching out to the authors or co-authors of all the studies included in our scoping review. We explained the scope of our research and requested an interview. The final number of interviews was based on the availability of expert interviewees. Additional experts were contacted using a snowball sampling technique.

### Data collection

We collected primary data by interviewing SSLE organizers and participants. Additional material was provided by interviewees. A female public health researcher (IT) conducted virtual semi-structured interviews from 1 ^st^ September 2021 to 26 ^th^ November 2021 via Google Meet. The semi-structured interviews were conducted in English. Notes were taken during the interviews. The researcher introduced herself, provided the full details of the study and requested verbal informed consent from all interviewees prior to initiating and recording the interview. IT has gained experience in qualitative research during her PhD and Post-doc in Healthcare Management, and she was providing technical support on the implementation and analysis of SSLEs at the WHO. Only one participant knew the researcher conducting the interview prior to the study. All interviewees were assured confidentiality: interviews were anonymised by assigning a number to each participant. There were no repeat interviews for the study. The interview guide was piloted during the first two interviewees. We transcribed the interviews verbatim. Transcripts were not returned to participants for their review and comments.

### Data analysis

We reviewed the transcripts and developed an initial extensive codebook (open coding), which enables the identification of emerging categories. This first round of coding was open-ended in a constant comparative process ( Braun & Clarke, 2006), after which the codebook was piloted on the first two transcripts and revised. Data were imported to a qualitative package RQDA (HUANG Ronggui (2016). RQDA: R-based Qualitative Data Analysis. R package version 0.2-8. http://rqda.r-forge.r-project.org/) that supports coding and data management. We reviewed the transcripts line-by-line and assigned codes. Categories were organized into two main themes (strengths and weaknesses) in order to answer the research question. We reviewed all the previous analysis, and sorted data to the point of saturation. Three authors coded the data with one completing the primary coding of the entire dataset (IT), which was reviewed by two researchers (RK and KA) to verify the soundness and completeness and add emerging codes. The themes were discussed and interpreted by IT and RK. Three co-authors (IT, RK and KA) addressed the organizational aspects of this study, the process of analysis and agreed on data saturation. IT, RK and JK interpreted the data.

### Ethical considerations

The WHO’s ethics review committee exempted this study from review (ERC.0003752). The study qualified for an exemption based on the Council for International Organizations of Medical Sciences (CIOMS) criteria and the WHO ERC RoP since: “public officials are interviewed in their official capacity on issues that are in the public domain”. Verbal informed consent for publication of the findings of this study and participants details was obtained.

## Results

### Expert characteristics

We interviewed sixteen experts from different countries and nationalities, with equal representation of female and male. Participants demonstrated varying levels of experience and performed various roles in the learning exchanges. Table 1 shows the characteristics of the sample. Three experts were not directly involved in SSLEs; however, they participated in various other research projects in collaboration with countries from the global south or in the development of the first draft of the SSLE guide. Time taken for interviews ranged from 30 to 60 minutes.

**Table 1.** Characteristics of study participants.

Expert	Name of the organization	Their experience in SSLE	Gender
1	United Nations Population Fund- UNFPA	Implementing partner / Facilitator	Male
2	Family Health International 360- FHI360	Facilitator	Female
3	Blue Ventures	Author of the guide on Fisheries Learning Exchanges	Male
4	Strategic Consulting for Health - Rising outcomes	Co-author of the WHO guide on SSLE	Female
5	United Nations Population Fund- UNFPA	Implementing partner / Facilitator	Male
6	Nahdlatul Ulama ^1^ - NU	Facilitator	Female
7	National Family Planning Coordinating Board- BKKBN	Facilitator	Male
8	Partners in Population And Development, Africa Regional Office- PPD	Facilitator	Female
9	Partners in Population And Development- PPD	Facilitator	Female
10	Blue Ventures	Facilitator	Male
11	United Nations Population Fund- UNFPA	Implementing partner / Facilitator	Male
12	United Nations Population Fund- UNFPA	Implementing partner / Facilitator	Female
13	Partners in Population And Development- PPD	Facilitator	Female
14	HERA, Right to Health and Development	Co-author of the WHO-guide on SS Cooperation	Male
15	World Health Organization- WHO	Scientist working in SRHR ^2^	Male
16	Knowledge Success	Facilitator	Female

^1^ Nahdlatul Ulama is the world's largest Muslim organization located in Indonesia ^2^ Sexual and Reproductive Health and Right

## Strengths

**Figure 1.**
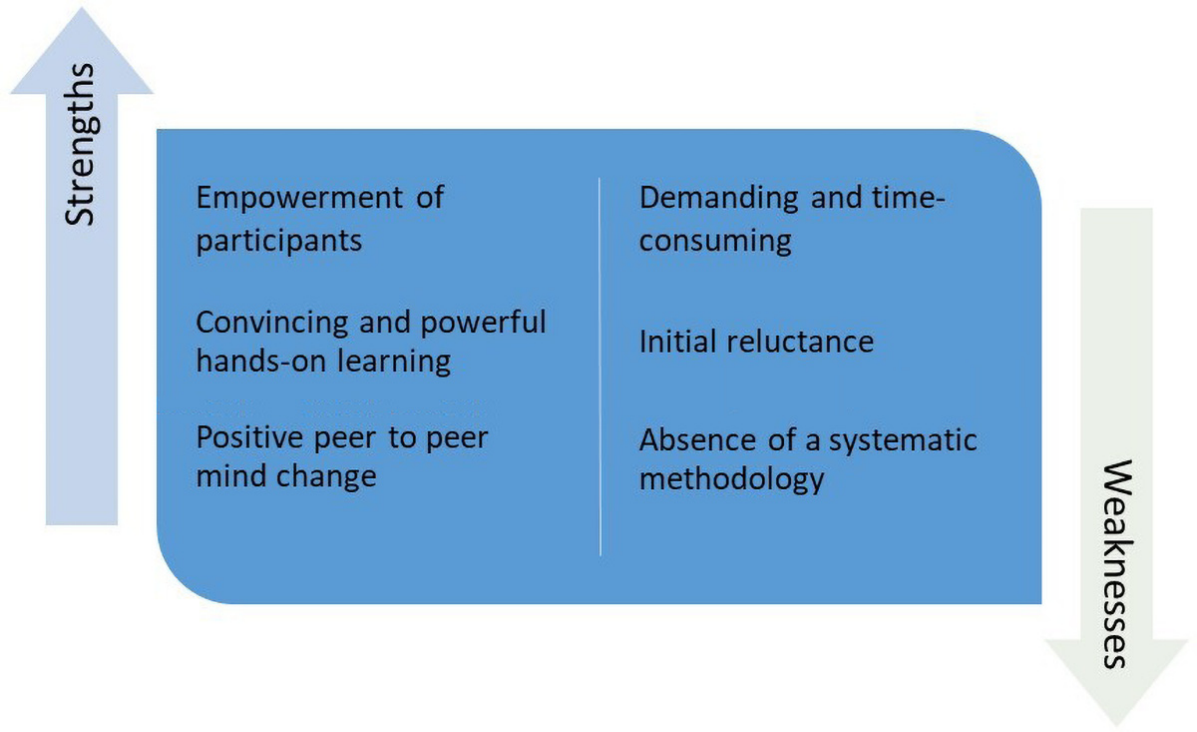
Strengths and weakness of SSLE.

We identified three main strengths associated with the SSLE process: empowerment of participants, positive peer-to-peer “mind change” and convincing and powerful hands-on learning ( Figure 1).

### Empowerment of participants

In a SSLE, participants are the key actors throughout the process; they identify the needs and purpose behind the exchange, outline the objectives, and conduct the exchange (Extended data- supplementary quotes, Table 1). Participants play a greater active role and are more likely to act on their own decisions. As reported by one facilitator, in some Muslim countries there were concerns on the compatibility of family planning with Islamic teachings, while other countries, like Indonesia, mobilized religious leaders to present their opinions and interpretations of the Islamic law. During an exchange on Family Planning among Muslim religious leaders between Indonesia and Chad, Expert 1 described the primary role of the team in advancing the exchange:

The teams navigate through Islamic law and how it interprets the use of contraceptive and family planning services. The teams set their goals. The facilitator leaves the team to move at their own pace without trying to shake them that much. (Expert 1)

Another example on the role of participants of SSLE is reported by Expert 2 who facilitated several study tours among various regions and countries. These SSLEs aimed at scaling up the use of injectables in the hardest-to-reach communities in African countries (for example, Rwanda, Uganda). The following quote described the key role of participants:

Part of the success of this process comes from individuals, connection, and passion around it. For example, the researcher I worked with, was really a mover-shaker, and he spread messages about the advantages of using injectables as a contraceptive. He was well connected with USAID that was also interested in the same activity. A study tour is successful when delegates, after reaching back home, make a presentation about it [their learnings during the exchange], write a brief, get in the agenda, talk to working group and create more awareness. (Expert 2)

Additionally, the facilitator supports the creation of a safe environment where team members share their own experiences. Discussions generate ideas and possible solutions, yet at the same time, they may be sensitive and lead to conflicts. Facilitators can often recognize these situations and steer around these challenges. This inclusive environment encourages diversity, connection, and relatedness. When participants feel connected, they are more prone to feel engaged and motivated, as described in the following quotes (Extended data- supplementary quotes, Table 1):

People [participants] do not know each other and sometimes come from different contexts. They will spend a lot of time together in quite a short period, so sometimes there are social barriers- the facilitators try to build empathy and trust. We facilitate them by accommodating visitors with a host family in our home rather than in a local guest house or hotel. Sometimes, the teams have low empathy and trust; we must recognize it and do what we can quickly. (…) A good facilitator is essential. (Expert 3)
  If they [both team] were not really excited by what they were doing, they would not be good mentors and mentees. It is not going to lead to a constructive learning exchange. (Expert 4)

The implementation of learnings strongly relies on team, participant and stakeholder interest and engagement. For example, Expert 5 reported that “90 out of 100 participants” attend an exchange in a passive way, and have no interest in implementing the learnings or advocating for a change upon returning to their team. Should these individuals be promoted or change jobs, the benefit of the learning exchange is lost. Experts suggested that a champion with a standing influence who makes the changes required is an enabling factor for an SSLE. The strength of champions is their empowerment and engagement.

### Positive peer-to-peer “mind change”

Exchanges with peers who are managing similar challenges are more likely to lead to a positive “mind change” and innovative solutions. Peers that face similar demographic, cultural and socio-economic characteristics provide more credible problem-solving models in comparison to those who are not coping with similar challenges. Expert 1 supported an exchange between Chad and Indonesia where Chad reports some of the highest maternal mortality rates in the world and 60%–70% of the population identifies as Muslim. Indonesia was selected as peer country since it runs successful SRHR programmes and is a Muslim-majority country. After this exchange, Expert 1 from Chad reported:

We were able to increase family planning acceptance. League of Women Preachers [a group of female Muslim teachers who are closely associated with Chad's Council of Islamic Affairs] works to push women to deliver at the health facilities. We cannot ask for more than that. At a slow pace, we can change their mindset. It is critical because it will last and these women will transfer new values to their children. (Expert 1)

A negative experience was reported by Expert 3 that facilitated an exchange between Mexican and Malagasy fishery communities. Despite the teams' dissimilar economic characteristics and needs, the SSLE was driven in response to pressure from donors. This difference affected the results of the SSLE and resulted in negative consequences.

There can be huge and unintended consequences to SSLE when the power and context is different between nations. The species cohort (octopus) were similar in Mexico and in Madagascar, but the standard of living was very different. The octopus fishers in Mexico, [have] comfortable standard of living – they have iPhone. In Madagascar they earn on average of 2 dollar a day (…). In this example, it was a double reciprocal exchange, the Malagasy fishers learned a new fishing method from their Mexican counterparts, which, if implemented in Madagascar, could negatively impact stocks there. (Expert 3)

Therefore, exchange with peers who share common characteristics is more likely to result in positive “mind changes”. Additional quotes can be found in *Extended data- supplementary quotes* (Table 2).

### Convincing and powerful hands-on learning

SSLE’s country tours and expert visits enables hands-on learning and for teams to explore and experience innovative solutions. This experience allows teams to engage authentically to prior knowledge and conceive new understandings. Participants are further encouraged to share and reflect on the learned diverse paradigmatic views to fully integrate ideas (Extended data- supplementary quotes, Table 3). Expert 6 provided an example where the seeker team implemented the learning after several discussions and debate. The discussion was about the gender relationship and how it translated into decision-making in the family:

I always see a change of paradigms, especially in Muslim-only workshops […]. Before the exchange, some of these Muslim religious leaders had very strong or ambiguous opinions about what Islam would say on certain topics. They [initially] opposed vasectomy and tubectomy as there is a law in Islam that says: you cannot make permanent change to your body. After this discussion and debate, they went to the field, and they got vasectomies in Indonesia. I do not think it was an easy decision for them, and that showed how big is the impact of this discussion. (Expert 6)

Due to the COVID-19 outbreak in March 2019, study tours and field visits were suspended. This impacted SSLE as travel to other country teams and accordingly learning by seeing was interrupted. Despite the inability to have face to face discussion, most SSLEs continued via online virtual platforms. Countries found innovative ways (conference calls using internet, zoom, WhatsApp, TEAMS etc) to work within and between countries with the added benefit of enabling more people to join the exchange and reducing the cost of the SSLE. However, some experts highlighted the added value of face-to-face exchange (or a combination of both virtual and in-person) compared to online meetings, in which teams may not fully understand how a practice has been implemented. In addition, in-person meetings facilitate connection and communication, establish a common ground for dialogue, and build solidarity and empathy at greater rates than in online virtual meetings.

## Weaknesses

We identified three main weaknesses associated with SSLEs: demanding and time-consuming process (human resources, working days, costs, logistics); participants initial reluctance to SSLE approaches; absence of a systematized and internationally recognized methodology.

### Demanding and time-consuming process

One of the main barriers identified by all the interviewed experts was that SSLEs require extensive resources and investment. A study tour or an expert visit requires time, funds, and human resources, as suggested by Expert 3 who was the moderator of several exchanges and oversaw their logistics (i.e. arranging flights and visas). Some experts faced several logistical issues when planning exchanges with either poor and marginalized communities or with countries currently experiencing humanitarian emergencies (conflict or war). As reported by the Indonesian Government (Expert 7), several tour visits were cancelled last minute due to conflicts or war within the country. Greater resources and more staff should be allocated to the exchanges, as illustrated in the following quote:

The main barriers are the cost - it is never cheap at all. Staff devoted to the visit exchange could not be devoted to actual conservation activities on the ground. Everyone must stop what they are doing for a week [duration of the visit exchange]. (Expert 3)

Additionally, the cost and resources spent on an exchange may not produce the expected results, as participants may change roles throughout the process and leave the programme prior to completion. Whenever experts work with policy makers to change policy, organizations keep engaging them and bringing them together. However, “they are very expensive people to maintain, and they keep changing. So, we may have been pursuing a policy initiative with a group of policy makers, then election coming, they lose the election and we have to start from the new ones” (Expert 8). Then, several experts reported that scarcity of resources delayed the planned activities, such as a training programme in Indonesia (Expert 7) or hindered exchanges and the follow-up activities (Expert 2 and 3) (Extended data- supplementary quotes, Table 4).

Despite requiring and utilising substantial resources, SSLE sustainability and cost-effectiveness may not be guaranteed. As reported by Expert 3, the facilitator had to convince a fishery community to participate in the exchange and had to compensate the community for the loss of value since they were not fishing during the visit exchange. Despite this, the exchange did not lead to the expected outcome - the community did not implement a conservation area. The benefit of the exchanges may not outweigh their costs.

Furthermore, interviewees highlighted that specific national South-South cooperation (SSC) budgets for SSLEs are often not provided by governments or international organizations. Exchanges are usually financed by organizations through their own funding or membership fees (Extended data- supplementary quotes- Table 4).

### Participant initial reluctance

Prior to participating in SSLEs, participants and stakeholders alike often demonstrate little interest in SSLE programs. SSLE champions need to actively advocate for an exchange program and its added benefits:

Six years ago, we started talking about family planning integration and we started implementing it in Nepal, but many governments did not want to attend meetings around it. Once we have showed them [the results of the exchange], the SSLE generated their interest. Meetings and international meetings, that bring experience from other regions, are key. (Expert 5)

Experts encouraged the inclusion of the skeptical participants into the exchange. Once they are engaged, they would be able to convince other skeptics. Expert 3 illustrated the benefits of engaging a skeptical community in the exchange between fishing communities on re-conservation in Madagascar:

Selecting people who were skeptical about the SSLE sounds stupid. (…) These people will often be the first to oppose a management or conservation measure, so turning them into early advocates is enormously advantageous. (Expert 3)

Apart from stakeholders and participants, a barrier faced by organizations in promoting SSLEs is the limited national political commitment to and operationalization of SSLE as an alternative model. In addition to that, expert 9 highlighted the absence of national policy and strategic frameworks, as well as absence of international coordination in South-South cooperation. New participants often do not have the motivation, time or willingness to take part in an exchange. Greater confidence and approaches from participants and governments may strengthen the SSLE’s organization and its operationalization.

### Absence of systematized and internationally recognized methodology

Nowadays, most organizations who gain experience in conducting SSLEs draft their own internal guide and tools on how to facilitate an exchange, as illustrated in the following quotes:

UNFPA has an internal guide document. The process can be divided into three distinct phases: 1) the consultation, 2) planning and implementation of activities; and 3) joint review of progress after one year. The Government commits to holding a consultation once a year. (Expert 1)
  The process is not the same for every exchange, that is why we tended to give a flexible 10-steps framework in the guide. It does not depend on the objectives. Generally, you must have three clear objectives you want to achieve, you do an informal M&E, debriefing and follow-up when we finished. Over the years, we developed templates to help manage the exchange. (Expert 3)

The SSLE process is not systematized and is not documented. Participants do not reserve time to write their learnings and discuss, even if this step is considered valuable and helps in growing and improving the participants skills and exchange results (Extended data- supplementary quotes, Table 5).

Regarding SSLE follow-ups (tracking results and reporting), the facilitator often conducts post evaluation, such as a follow-up survey or informal feedback from the participants, often in a unstandardised way (Extended data- supplementary quotes- Table 5). Regular follow-up meetings can facilitate and support the implementation of action plans, but several experts declared that these meetings are rarely organized due to budget constraints and limited interest of participants.

During the SSLE, most experts developed an action plan or roadmap for implementing the know-how in their country/community and few of them monitored the process after exchange. The Government of Indonesia, cooperating closely with Southern partner since 1955, has established a methodology and developed tools to annually report and evaluate all the activities during and after the SSTC programmes. Other organizations have developed their internal tools, as demonstrated in the following quotes:

During the exchange with the Philippines, we developed a five-year roadmap. Then, we evaluated [the progress] yearly because we held the steering committee meeting a year afterwards, where we evaluated last year's program and developed next year's program. (Expert 11)

Even if each organization developed its instruments and tools for implementing the gained knowledge, all the experts highlighted that monitoring and evaluating (M&E) is challenging and it is often omitted, as illustrated in the following quote:

The monitoring focused on the action plan, rather than monitoring progress post-broader M&E. (Expert 4)

## Discussion

This study sheds light on some strengths and weaknesses of South-South learning exchanges by examining the perspective of a range of experts from different disciplines. Empowerment of participants, positive peer-to-peer “mind change”, powerful knowhow are the main strengths of the SSLE approach. Resource heavy, participants reluctancy and absence of a validated guide methodology emerged as main weaknesses of SSLE, which could impair the effectiveness of this approach. This study illustrated that SSLE is a promising and valuable tool to pass on knowledge and information from a grass-root approach.

The SSLEs’ strengths derived from two main processes previously described in the management, educational and psychological literature: experiential learning and social learning.

SSLE provides participants with hands-on, personal experiences, that are key to experimental learning. As described in the Kolb’s experimental learning model ( Kolb, 2015) and its subsequent revision ( Morris, 2019), learning consists of a four-stage cycle that includes a concrete experience, a reflective observation on the experience, conceptualization, and an active experimentation of what you have learnt. “Learning by doing” has been highlighted as a funding concept of this approach by Morrison *et al.* ( Morris, 2019). Learners are immersed in this learning experience that contains context-specific information, and this “hands-on” process makes learners active and empowered, as corroborated by the identified strengths. In fact, participants’ reflection on the acquired knowledge or experience, internalization and application of the knowhow are the strengths of the South-South model. This approach leads to an understanding that is different from that acquired through research, observation, books, and lessons ( Borkman, 2007). Moreover, these “hands-on” process enhances ownership, enthusiasm and leadership among participants and stakeholders.

During the exchange, participants are involved in the process of social learning ( Schusler *et al.*, 2003). The social learning theory postulated that “social behaviour is learned by observing and imitating the behaviour of others” and suggested that “behaviour change is more likely when modelling is provided by peers than non-peers” ( Bandura & RH, 1977). Interactions with peers who are successfully coping with their experiences are more likely to result in positive behaviour change and peers are more credible role models for others. Personal interactions created a common understanding and may encourage the continuous sharing of best practices after the exchange event ( Jenkins *et al.*, 2017). This study showed that participants and stakeholders from countries with similarity exchange knowledge and expertise in a convincing way, such as the exchange run among Indonesian and Chadian Muslim religious leaders on family planning. Peer model is a key strength of the SS learning exchange.

As SSLEs are demanding and time-consuming, they require considerable investment by facilitators and participants. Previous literature confirmed that traditional exchanges are extremely costly and time intensive ( Gardner *et al.*, 2017; Jenkins *et al.*, 2017). A recent experience conducted by the co-authors demonstrated SSLE can be successfully run using an online platform ( Kabra *et al.*, 2022) with lower costs and resources. However, the interviewees reported that the virtual modality is less powerful than face-to-face sessions where participants can experience hands-on learning, as suggested by the experiential learning model. As these exchanges require significant investment to achieve desirable results, national and international commitment and funding are key to operationalising these exchanges.

The participants' and government’s reluctance to support the SSLE approach can be explained by the limited evidence on the impact of the exchanges and the implementation of learnings ( Olu *et al.*, 2017). Most SSLE programmes are small, informal or nested in cooperation programmes ( WHO & World Health Organization, 2014), and the documentation and dissemination of results are undervalued. Our findings showed that, when in place, the monitoring, evaluation, and follow-up processes are perceived as blended or inappropriate, that can lead to further resistance and reluctance. The inappropriateness of these processes is highlighted in the “Formative evaluation of UNFPA approach to South-South and triangular cooperation” ( UNFPA Evaluation Office, 2020) and by other scholars, such as Jenkins *et al.* and Thompson *et al.* ( Jenkins *et al.*, 2017; Thompson *et al.*, 2017). Nevertheless, there are exceptions to the trend; for example, the Korean Development Institute and World Bank Institute assessed the knowledge exchange programs using a results-focused methodology in three case studies ( Chun & Kim, 2011). Likewise, Nepal and Sri Lanka teams developed a monitor tracker and framework to implement the action plan during a SSLE facilitated by the WHO ( Kabra *et al.*, 2022). These exceptions show that a robust method (including the process of documenting and M&E) can lead to a more accurate demonstration of the impacts of SSLE, increased credibility, visibility to participants, governments, and funding agencies, then improved availability of and accessibility to learning on the SSLE.

We note several limitations of this study. Participants were selected from available SSLE organizers or participants contacted by the authors of this study. There were challenges in recruiting interviewees since we were solely able to contact them by email. Finally, recruitment was done by the authors of this study who work at the SRHR department, and several interviewees were SRHR experts, there may be some specific bias in recruitment.

To our knowledge, this is the first manuscript exploring the perspectives of a sample of experts on SSLE from different disciplines. The originality of this study lies in the collection of various personal and professional SSLE experiences: we sampled across various professional levels (government representatives, NGOs, communities) and captured the far-ranging capacities of SSLE application (from fishery management to family planning). An additional strength of this study stems in part from the interdisciplinary team. Co-authors of this study are from different disciplines such as healthcare management, public health, human reproduction and family planning.

This manuscript demonstrated the need for further evidence on the SSLE effectiveness. Future research should also consider the long-term impact of the SSLE and its sustainability.

## Conclusion

SSLE is a promising approach to exchanging knowledge and experience among peers. A robust and recognized methodology within all process phases is likely to help achieve the expected results, increase available evidence and demonstrate its success. However, this approach requires the support of national funds to improve its availability and accessibility.

## Data Availability

In order to protect the privacy of the participants, the full data containing identifiable information has not been made publicly available. However, researchers in a related field can request additional details about the interviewees. To obtain the data, interested researchers should send an email to isotta.triulzi@santannapisa.it with the subject line 'Strengths and weaknesses of SSLE' and explain their reason for needing the data. They must also confirm that the data will not be made public or misused, and that the sharing is documented. Repository: *Experts’ perspectives on strengths and weaknesses of the South- South Learning Exchange: a qualitative analysis*. DOI: 10.6084/m9.figshare.22742180 This project contains the following extended data: Interview Guide Supplementary Quotes Data are available under the terms of the Creative Commons Zero "No rights reserved" data waiver (CC0 1.0 Public domain dedication). Repository: Consolidated Criteria for Reporting Qualitative Research (COREQ) check list for *Experts’ perspectives on strengths and weaknesses of the South- South Learning Exchange: a qualitative analysis. DOI: 10.6084/m9.figshare.23045015* Data are available under the terms of the Creative Commons Zero "No rights reserved" data waiver (CC0 1.0 Public domain dedication).
