# Strengths and weaknesses of the South-South Learning Exchange: a qualitative analysis of experts’ perspectives

**DOI:** 10.12688/gatesopenres.14699.2

**Published:** 2024-03-27

**Authors:** Isotta Triulzi, Rita Kabra, Komal Preet Allagh, James Kiarie

**Affiliations:** 1 Institute of Management, Scuola Superiore Sant'Anna, Pisa, 56127, Italy; 2 Department of Sexual and Reproductive Health and Research, World Health Organization, Geneva, 1211, Switzerland; 3 Consultant, Department of Sexual and Reproductive Health and Research, World Health Organization, Geneva, 1211, Switzerland

**Keywords:** learning exchange; South-South learning exchange; peer-to-peer; South-South cooperation; knowledge exchange

## Abstract

**Background:**

South-South learning exchange (SSLE) refers to an interactive learning process where peers exchange knowledge and experience to work towards a beneficial change. Despite organizations having recently increased the opportunity to run SSLEs, the SSLE support mechanisms and processes are not well documented in the scientific literature. This study explored experts’ perspectives on SSLEs, strengths, weaknesses and mechanisms leading to sustainable outcomes.

**Methods:**

We conducted a qualitative study using semi-structured interviews on experiences of participants and organizers of SSLEs. Data were collected between 1st September 2021 to 26th November 2021. All data were digitally recorded, transcribed verbatim, and analysed. In the analysis, we adopted an inductive approach derived from thematic analysis.

**Results:**

Sixteen experts, who have participated in or facilitated one or more SSLE, were interviewed. The experts’ accounts demonstrated an appreciation of participants’ empowerment, positive peer-to-peer “mind change” and convincing and powerful hands-on learning of this approach as strengths in the implementation of the SSLE. Being resource heavy, participant and donor reluctance and absence of a validated methodology emerged as main weaknesses of the South-South learning approach, which could impair the effectiveness of this scheme.

**Conclusions:**

The strengths of SSLEs are anchored in the theories of experiential and social learning, highlighting SSLE's potential to create an environment that enhances knowledge exchange. the study highlights the challenges SSLE initiatives face. In particular, these include limited commitment and funds, limited evidence of impact, disparate approaches, and the absence of standardized guidelines and evaluation practices.

## Introduction

The South-South learning exchange or peer-peer learning exchange refers to an interactive learning process where peers exchange knowledge and experience to work towards a beneficial change ( WHO, 2018a). These exchanges are often referred as south-to-south cooperation (SSC) or knowledge exchange. The WHO’s thirteenth “General Programme of Work (2019–2023)” ( World Health Organization, 2018b) integrated south-to-south cooperation as a strategy to develop and scale up innovative solutions for building capacities through shared learning and equitable partnerships, thereby contributing to the achievement of the Sustainable Development Goals. Some authors argued that the concept of SSC lacks a universally accepted definition. This absence of consensus is driven by the ongoing discussion around the nuances of "southern" identity, the multifaceted nature of "development", and the dynamics of "cooperation" — whether it occurs among equals or otherwise ( Avis, 2022; Besharati & MacFeely, 2019; Caixeta & dos Santos, 2022). This complexity underscores the challenge in creating one definition of SSC and SSLE, reflecting the diverse experiences and expectations of the actors involved in south-to-south cooperation.

Although the debate on common definitions is ongoing, SSLEs have some key aspects in common. The exchanges allow participants to “learn firsthand from the experience of their peers how a challenge was solved or solution implemented” ( Kumar & Watkins, 2017). The SSLE can inspire peers, create new ideas, implement reforms, share practical problem-solving ‘how-to’ knowledge, and foster collaboration and advocacy ( The World Bank, 2019a) – all key factors for social learning processes ( Reed *et al.*, 2010). They require coordination with two peer teams (knowledge seeker and knowledge provider), a facilitator (broker) who brings the two teams together, and various stakeholders that support the work towards a change, or have an interest in the outcomes of the SSLE ( World Health Organization, 2018b). Organizers are usually international agencies, non-governmental organizations (NGOs) and governments (e.g. India, South Africa, China, Brazil) and the SSLE can be bilateral, multilateral, intraregional or interregional. There isn't a universally set duration, and flexibility allows adaptation to specific needs.

Governments, agencies and institutions have increased over time the opportunity to run SSLE programs to share practices and experiences in various ways (i.e. visit tours, platforms) ( Food and Agriculture Organization of the United Nations (FAO), 2021) and areas, such as development, climate change, conservation management, and reproductive health and rights ( Partners In Population And Development, 2021; The World Bank, 2019a). Previous peer-reviewed literature reporting SSLE experiences is limited to specific topics such as conservation management ( Bretos *et al.*, 2017; Gardner *et al.*, 2017; Heyman & Stronza, 2011), information system in health ( Were *et al.*, 2019), human capacity on HIV/AIDS services ( Ivers *et al.*, 2010), disease outbreak and health system strengthening ( Olu *et al.*, 2017). Grey literature illustrated results of SSLEs conducted by several agencies, such as the World Bank ( The World Bank, 2019b), the United Nations Development Programme ( United Nations Development Programme, 2017), the United Nations Office for South-South Cooperation ( United Nations Office for South-South Cooperation, 2019; United Nations Office for South-South Cooperation, 2020), and the United Nations Population Fund ( United Nations Population Fund (UNFPA), 2018; United Nations Population Fund (UNFPA), 2021). The increasing adoption of SSLE programs by various entities reflects a growing recognition of its value across diverse fields, and positions SSC as a critical means for the Global South to innovate and decolonize south-south cooperation and development, as well as fostering a new identity within the realm of international politics ( Caixeta & dos Santos, 2022; Waisbich, 2022). Despite scholars pointing out the challenges faced in SSC, discussing the need for defining and whether or how to measure SSC ( Besharati & MacFeely, 2019; Waisbich, 2022), the peer-reviewed literature on SSLE initiatives remains scarce. This highlights the need for academia to have access to information on SSLE to conduct more in-depth analyses to inform, support, and evaluate policy-making.

The WHO embarked on the Family Planning (FP) Accelerator project in 2019 ( World Health Organization, 2020) with the objective to improve access to quality and rights-based FP services. Under this project, ten (five bilateral SSLE) low-income countries have participated in SSLE. These exchanges follow a five-step approach using the preliminary version of “A step-by-step Guide to South–South learning exchanges” guide ( World Health Organization, 2018a), which allows the planning, conducting and evaluation of an SSLE to plan, conduct, and evaluate a SSLE. The steps include defining the need for and the purpose of the learning exchange; planning and facilitating the learning exchange; supporting implementation of the action plan and following-up after the learning exchange. An example is the SSLE between the Nepalese and Sri Lankan ministries of health, in which respective teams shared their best practices and learnings. This resulted in the implementation of a web-based system for logistics management of FP commodities in Sri Lanka and Nepal. They started the implementation of integrated family planning services in a decentralized environment, using a lifecycle approach to improve the uptake of postpartum FP ( Kabra *et al.*, 2022). The strengths of the WHO approach laid on the strong emphasis on the preparation phase and prioritization of the learning question before the exchange begins. This methodology gives considerable attention to the phases following the exchange. This includes post-exchange implementation, follow-up, and comprehensive documentation of the outcomes and lessons learned.

The authors of this study conducted a scoping review of both published and grey literature on SSLE that included 29 articles, where 27 were reports, case studies or press releases and only two peer-reviewed publications ( Allagh *et al.*, 2023). This review captures four types of approaches adopted for conducting SSLEs: study tours (reciprocal, non-reciprocal), virtual exchanges, expert visits, and mixed method exchanges (study tour and expert visits; virtual reciprocal exchange), where a study tour is the most common approach. Policy dialogue was identified as the primary output of these SSLEs and improved contraceptive prevalence, which is the most frequently reported outcome. Ambiguity remains regarding the extent to which SSLEs have contributed to FP outcomes due to the limited reported evidence and consistent documentations (i.e., information on implementation of learnings, documentation of lessons learnt, information on selection of participants), and their scarce quality (i.e., evaluation of SSLE and documentation of results). Hence, it is evident that there is a compelling need to systematize the SSLE approach.

By conducting this qualitative analysis of experts' perspectives, this paper aims to shed light on the strengths and weaknesses of the South-South Learning Exchange approach. The insights gained from this study will contribute to the existing knowledge and inform future efforts to enhance the impact of SSLEs.

## Methods

### Design

We conducted a qualitative study using key informant interviews to explore the experience of participants and organizers of SSLEs. We adopted an inductive approach derived from thematic analysis. This qualitative study follows the “Consolidated Criteria for Reporting Qualitative Research COREQ” ( Tong *et al.*, 2007) (Extended data).

### Research setting

This qualitative study is embedded into the WHO Family Planning Accelerator project, in which countries adopted a South-South learning approach. The project is overseen by the Contraceptive Unit at the Department of Sexual Reproductive Health and Rights at the WHO. The authors of this study have recently conducted a scoping review on the purposes, approaches, barriers, facilitators and outcomes of SSLE in FP ( Allagh *et al.*, 2023), which follows the six-step methodological framework suggested by Levac *et al.* ( Arksey & O’Malley, 2005; Levac *et al.*, 2010). The last step of this framework includes a “stakeholder consultation” that was preceded by the key informant interviews with the experts analysed in this paper. This analysis was the basis for the stakeholder consultation held at the WHO in November 2021, where experts shared their experiences, enablers, barriers and lessons learnt with the goal of making future SSLEs more efficient and effective. All the interviewers were invited to the 2021 WHO stakeholder consultation.

### Interviews

We conducted semi-structured interviews using a pre-structured interview guide (Extended file- interview guide), with open-ended questions that covered the following areas:

I. Process and methodology adopted during the SSLEs;
II. Participants’ personal and professional experiences on SSLE programs, including challenges, enablers, and lessons learnt.

We sampled purposively by reaching out to the authors or co-authors of all the studies included in our scoping review and reports or publications identified in preliminary research on SSLE. We explained the scope of our research and requested an interview. The final number of interviews was based on the availability of expert interviewees. Additional experts were contacted using a snowball sampling technique.

### Data collection

We collected primary data by interviewing SSLE organizers and participants. Additional material was provided by interviewees. A female public health researcher (IT) conducted virtual semi-structured interviews from 1 ^st^ September 2021 to 26 ^th^ November 2021 via Google Meet. The semi-structured interviews were conducted in English. Notes were taken during the interviews. The researcher introduced herself, provided the full details of the study and requested verbal informed consent from all interviewees prior to initiating and recording the interview. IT has gained experience in qualitative research during her PhD and Post-doc in Healthcare Management, and she has provided technical support on planning, implementation and monitoring of various SSLEs (including the SSLE between Sri Lanka and Nepal in the introduction) within the Family Planning (FP) Accelerator Project at the WHO for almost two years.

Only one participant knew the researcher conducting the interview prior to the study. All interviewees were assured confidentiality: interviews were anonymised by assigning a number to each participant. There were no repeat interviews for the study. The interview guide was piloted during the first two interviewees, adapted and a final version was developed. We transcribed the interviews verbatim. Transcripts were not returned to participants for their review and comments.

### Data analysis

We reviewed the transcripts and developed an initial extensive codebook (open coding), which enables the identification of emerging categories. This first round of coding was open-ended in a constant comparative process ( Braun & Clarke, 2006), after which the codebook was piloted on the first two transcripts and revised. Data were imported to a qualitative package RQDA (HUANG Ronggui (2016). RQDA: R-based Qualitative Data Analysis. R package version 0.2-8. http://rqda.r-forge.r-project.org/) that supports coding and data management. We reviewed the transcripts line-by-line and assigned codes. Categories were organized into two main themes (strengths and weaknesses) in order to answer the research question. We reviewed all the previous analysis, and sorted data to the point of saturation. Three authors coded the data with one completing the primary coding of the entire dataset (IT), which was reviewed by two researchers (RK and KA) to verify its soundness and completeness and add emerging codes. Where disagreement or challenges arose, the reviewers consulted a third reviewer (JK) to reach a consensus. The themes were discussed and interpreted by IT and RK. Three co-authors (IT, RK and KA) addressed the organizational aspects of this study, the process of analysis and agreed on data saturation. IT, RK and JK interpreted the data.

### Ethical considerations

The WHO’s ethics review committee exempted this study from review (ERC.0003752). The study qualified for an exemption based on the Council for International Organizations of Medical Sciences (CIOMS) criteria and the WHO ERC RoP since: “public officials are interviewed in their official capacity on issues that are in the public domain”. Verbal informed consent for publication of the findings of this study and participants’ details was obtained

## Results

### Expert characteristics

We interviewed sixteen experts from different countries and nationalities, with nine experts from the Global South and seven from the Global North. Most participants were senior professionals and demonstrated varying levels of experience and performed various roles in the learning exchanges. Table 1 shows the characteristics of the sample. Three experts were not directly involved in SSLEs; however, they participated in various other research projects in collaboration with countries from the Global South or in the development of the first draft of the SSLE guide. Time taken for interviews ranged from 30 to 60 minutes, with an average length of 50 minutes.

**Table 1.** Characteristics of study participants.

No. Expert	Name of the organization	Experience/ role in SSLE	Gender	Country of origin	Year of Experience in conducing SSLE	Countries involved in SSLEs	Thematic area	Main participants	Type of SSLEs
1	United Nations Population Fund (UNFPA)	Implementing partner / Facilitator	Male	Chad	>5 years	Chad – Indonesia Extended to other African countries	FP, Muslim religion	Governments, UNFPA Academy, League of Women Preachers (Chad), Council of Islamic affairs of Chad, Muslim religious leaders, HCWs	Study tours (reciprocal exchange), congress exchanges (not include site visits)
2	Family Health International 360 (FHI360)	Facilitator	Female	US	>5 years	Uganda - Kenya, Nigeria, Rwanda. Extended to African countries	SRHR ^1^	Governments, implementing partners, USAID, HCWs, communities	Study tours (reciprocal and non-reciprocal)
3	Blue Ventures	Facilitator	Male	UK	>5 years	Madagascar - Mexico and other countries (i.e. Mauritius, Mozambique)	Fishery management, marine conservation	Fishing communities, NGOs, Governments	Study tours (reciprocal exchange), congress exchanges
4	Consulting company	Monitoring, evaluation, planning and implementation; co-author of the WHO SSLE guidelines	Female	US	<5 years	na	SRHR	na	na
5	UNFPA - Asia Pacific Regional Office	Implementing partner / Facilitator	Male	India	>5 years	Indonesia- Philippines; Korea- India Several other countries	SRHR, Muslim religion, capacity building Logistic Information Management system	Governments (i.e BKKBN, Commission on Population of the Republic of Philippines), NGOs, Muslim religious leaders	Expert visits, virtual exchanges, mixed method exchanges
6	Nahdlatul Ulama ^2^ - NU	Facilitator	Female	Indonesia	>5 years	Indonesia – various countries	SRHR , Muslim religion	UNFPA, Governments (i.e. BBKBN)	Study tours, short and intensive training workshops
7	National Family Planning Coordinating Board (BKKBN)	Facilitator	Male	Indonesia	>5 years	Indonesia – various countries	SRHR	Governments, NU, Muslim religious leaders, UNFPA	Study tours, virtual exchanges, expert visits, mixed method exchanges
8	Partners in Population and Development ^3^(PPD)-Africa Regional Office	Facilitator (organizing and coordinating SSC)	Female	Uganda	>5 years	Member countries from Africa (EAPACOH ^4^ and EARHN ^5^) (e.i. Uganda - Ghana, Kenya, Zambia)	Population and development, policy development	Governments, institutions, civil society	Virtual exchanges, workshops
9	PPD	Facilitator	Female	Bangladesh	>5 years	Member countries from Asia, Africa, Middle-East and Latin- America, China	Population and development, policy development	Governments (i.e. MoH), institutions (i.e. National population counsel), civil society	Training workshops, scholarship programme for government officials, study tours
10	Blue Ventures	Facilitator	Male	Europe	>5 years	Madagascar - several countries	Marine conservation, fishery management and FP	Fishers, fishery managers, NGOs (i.e. Marie Stopes International, Population service international), agencies (i.e. USAIDS, IPPF ^6^), governments	Expert visits
11	UNFPA	Implementing partner / Facilitator (responsible of SSC for the Government of Indonesia)	Male	Indonesia	>5 years	Indonesia- several countries (i.e. Philippines, Nepal, Ghana, Mali)	SRHR	Governments (i.e. Minister of Foreign affairs, State Secretariat, Religious Affairs, BKKBN), UN, academic institute, National Population and FP Board (NPFPC)	Training workshops, study tours
12	UNFPA- Pacific Sub-regional Office	Implementing partner / Facilitator	Female	New Zealand	<5 years	Fiji- Samoa	Maternal health services, disease outbreak	Retired Fijian midwives, Samoa HCWs, governments, emergency medical team	Expert visits
13	PPD	Facilitator	Female	India	>5 years	Member countries from Asia, Africa, Middle-East and Latin- America, China (i.e. Nigeria - Bangladesh)	Population and development	Governments (i.e. Ministers of Health, Finance, Social Development), UN agencies, International Organizations, donors, relevant stakeholders and development partners	Training workshops, scholarship programme for government officials
14	Independent cooperative company	Consultant, co-author of the guide on SSC (WHO, Word Bank)	Male	Europe	<5 years	Latin America, Cuba	Maternal and Child Health, Infectious diseases	PAHO ^7^, IDRC ^8^, and various academic institutions	Multiple projects featuring a triangular cooperation component
15	WHO	Scientist	Male	South Africa	>5 years	African countries	Multi-country qualitative research in SRHR	Various academic institutions	Unified research protocol, data analysis plans, codebooks shared among countries
16	Johns Hopkins Center for Communication Programs (Knowledge Success)	Facilitator	Female	US	<5 years	Partners of this programme (i.e. Senegal- Chad)	Population, Health, and environment, health financing	FP2020, Government (MoH), WHO representatives, USAIDS	Virtual exchanges

^1^Sexual and Reproductive Health and Right ^2^Nahdlatul Ulama is the world's largest Muslim organization located in Indonesia ^3^PPD is an intergovernal organization for promoting SS Cooperation https://www.partners-popdev.org/ ^4^Network of African Parliamentary Committees of Health ^5^Eastern Africa Reproductive Health Network ^6^International Planned Parenthood Federation ^7^Pan American Health Organization ^8^International Development Research Centre

## Strengths

**Figure 1.**
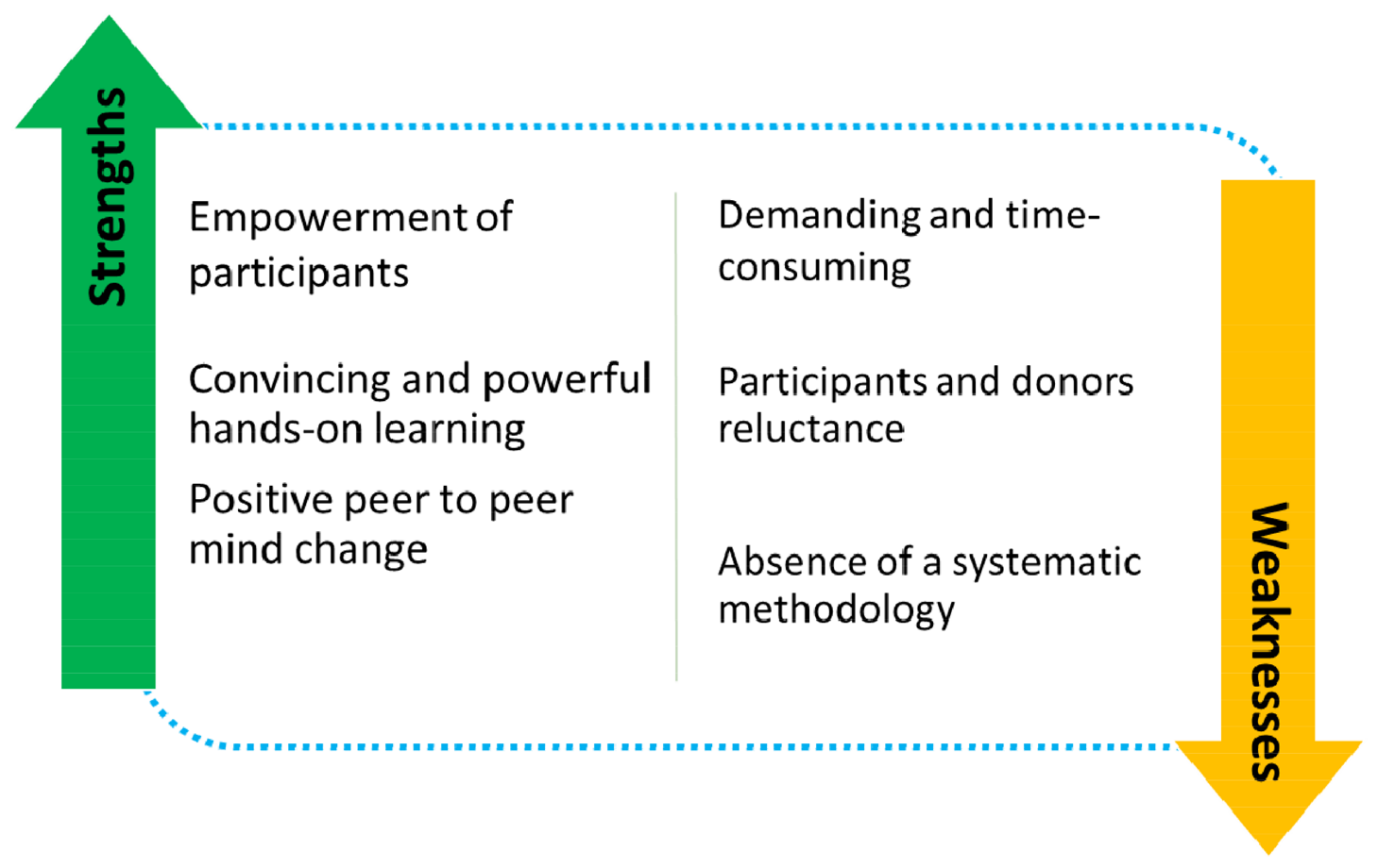
Strengths and weakness of SSLE.

We identified three main strengths associated with the SSLE process: empowerment of participants, positive peer-to-peer “mind change” and convincing and powerful hands-on learning ( Figure 1).

### Empowerment of participants

In a SSLE, participants are the key actors throughout the process; they identify the needs and purpose behind the exchange, outline the objectives, and conduct the exchange (Extended data- supplementary quotes, Table 1). Participants play a greater active role and are more likely to act on their own decisions. As reported by one facilitator, in some Muslim countries there were concerns regarding the compatibility of family planning with Islamic teachings, while other countries, like Indonesia, mobilized religious leaders to present their opinions and interpretations of Islamic law. During an exchange on Family Planning among Muslim religious leaders between Indonesia and Chad, Expert 1 described the primary role of the team in advancing the exchange:

The teams navigate through Islamic law and how it interprets the use of contraceptive and family planning services. The teams set their goals. The facilitator leaves the team to move at their own pace without trying to shake them that much. (Expert 1)

Another example on the role of participants of SSLE is reported by Expert 2 who facilitated several study tours among various regions and countries. These SSLEs aimed at scaling up the use of injectables in the hardest-to-reach communities in African countries (for example, Rwanda, Uganda). The following quote described the key role of participants:

Part of the success of this process comes from individuals, connection, and passion around it. For example, the researcher I worked with, was really a mover-shaker, and he spread messages about the advantages of using injectables as a contraceptive. He was well connected with USAID that was also interested in the same activity. A study tour is successful when delegates, after reaching back home, make a presentation about it [their learnings during the exchange], write a brief, get in the agenda, talk to working group and create more awareness. (Expert 2)

Additionally, the facilitator supports the creation of a safe environment where team members share their own experiences. Discussions generate ideas and possible solutions, yet at the same time, they may be sensitive and lead to conflicts. Facilitators can often recognize these situations and steer around these challenges. This inclusive environment encourages diversity, connection, and relatedness. When participants feel connected, they are more prone to feel engaged and motivated, as described in the following quotes (Extended data- supplementary quotes, Table 1):

People [participants] do not know each other and sometimes come from different contexts. They will spend a lot of time together in quite a short period, so sometimes there are social barriers- the facilitators try to build empathy and trust. We facilitate them by accommodating visitors with a host family in our home rather than in a local guest house or hotel. Sometimes, the teams have low empathy and trust; we must recognize it and do what we can quickly. (…) A good facilitator is essential. (Expert 3)
If they [both teams] were not really excited by what they were doing, they would not be good mentors and mentees. It is not going to lead to a constructive learning exchange. (Expert 4)

The implementation of learnings strongly relies on team, participant and stakeholder interest and engagement. For example, Expert 5 reported that “90 out of 100 participants” attend an exchange in a passive way, and have no interest in implementing the learnings or advocating for a change upon returning to their team. Should these individuals be promoted or change jobs, the benefit of the learning exchange is lost. Experts suggested that a champion with a standing influence who makes the changes required is an enabling factor for an SSLE. The strength of champions is their empowerment and engagement.

### Positive peer-to-peer “mind change”

Exchanges with peers who are managing similar challenges are more likely to lead to a positive “mind change” and innovative solutions. Peers that face similar demographic, cultural and socio-economic characteristics provide more credible problem-solving models in comparison to those who are not coping with similar challenges. Expert 1 supported an exchange between Chad and Indonesia where Chad reports some of the highest maternal mortality rates in the world. 60%–70% of its population identifies as Muslim therefore Indonesia was selected as peer country since it runs successful SRHR programmes and is also a Muslim-majority country. After this exchange, Expert 1 from Chad reported:

We were able to increase family planning acceptance. League of Women Preachers [a group of female Muslim teachers who are closely associated with Chad's Council of Islamic Affairs] works to push women to deliver at the health facilities. We cannot ask for more than that. At a slow pace, we can change their mindset. It is critical because it will last and these women will transfer new values to their children. (Expert 1)

A negative experience was reported by Expert 3 that facilitated an exchange between Mexican and Malagasy fishery communities. Despite the teams' dissimilar economic characteristics and needs, the SSLE was driven in response to pressure from donors. This difference affected the results of the SSLE and resulted in negative consequences.

There can be huge and unintended consequences to SSLE when the power and context is different between nations. The species cohort (octopus) were similar in Mexico and in Madagascar, but the standard of living was very different. The octopus fishers in Mexico, [have] comfortable standard of living – they have iPhone. In Madagascar they earn on average of 2 dollar a day (…). In this example, it was a double reciprocal exchange, the Malagasy fishers learned a new fishing method from their Mexican counterparts, which, if implemented in Madagascar, could negatively impact stocks there. (Expert 3)

Therefore, exchange with peers who share common characteristics is more likely to result in positive “mind changes” ( *Extended data- supplementary quotes*- Table 2)

### Convincing and powerful hands-on learning

SSLE’s country tours and expert visits enable hands-on learning and for teams to explore and experience innovative solutions. This experience allows teams to engage authentically to prior knowledge and conceive new understandings. Participants are further encouraged to share and reflect on the learned diverse paradigmatic views to fully integrate ideas (Extended data- supplementary quotes, Table 3). Expert 6 provided an example where the seeker team implemented the learning after several discussions and debate. The discussion was about the gender relationship and how it translated into decision-making in the family:

I always see a change of paradigms, especially in Muslim-only workshops […]. Before the exchange, some of these Muslim religious leaders had very strong or ambiguous opinions about what Islam would say on certain topics. They [initially] opposed vasectomy and tubectomy as there is a law in Islam that says: you cannot make permanent change to your body. After this discussion and debate, they went to the field, and they got vasectomies in Indonesia. I do not think it was an easy decision for them, and that showed how big is the impact of this discussion. (Expert 6)

Due to the COVID-19 outbreak in March 2020, study tours and field visits were suspended. This impacted SSLE as travel to other country teams and accordingly learning by seeing was interrupted. Despite the inability to have face to face discussion, most SSLEs continued via online virtual platforms. Countries found innovative ways (conference calls using internet, Zoom, WhatsApp, TEAMS etc) to work within and between countries with the added benefit of enabling more people to join the exchange and reducing the cost of the SSLE. However, some experts highlighted the added value of face-to-face exchange (or a combination of both virtual and in-person) compared to online meetings, in which teams may not fully understand how a practice has been implemented. In addition, in-person meetings facilitate connection and communication, establish a common ground for dialogue, and build solidarity and empathy at greater rates than in online virtual meetings.

## Weaknesses

We identified three main weaknesses associated with SSLEs: demanding and time-consuming process (human resources, working days, costs, logistics); participants initial reluctance to SSLE approaches; absence of a systematized and internationally recognized methodology.

### Demanding and time-consuming process

One of the main barriers identified by all the interviewed experts was that SSLEs require extensive resources and investment. A study tour or an expert visit requires time, funds, and human resources, as suggested by Expert 3 who was the moderator of several exchanges and oversaw their logistics (i.e. arranging flights and visas). Some experts faced several logistical issues when planning exchanges with either poor and marginalized communities or with countries currently experiencing humanitarian emergencies (conflict or war). As reported by the Indonesian Government (Expert 7), several tour visits were cancelled last minute due to conflicts or war within the country. Greater resources and more staff should be allocated to the exchanges, as illustrated in the following quote:

The main barriers are the cost - it is never cheap at all. Staff devoted to the visit exchange could not be devoted to actual conservation activities on the ground. Everyone must stop what they are doing for a week [duration of the visit exchange]. (Expert 3)

Additionally, the cost and resources spent on an exchange may not produce the expected results, as participants may change roles throughout the process and leave the programme prior to completion. Whenever experts work with policy makers to change policy, organizations keep engaging them and bringing them together. However, “they are very expensive people to maintain, and they keep changing. So, we may have been pursuing a policy initiative with a group of policy makers, then election coming, they lose the election and we have to start from the new ones” (Expert 8). Then, several experts reported that scarcity of resources delayed the planned activities, such as a training programme in Indonesia (Expert 7) or hindered exchanges and the follow-up activities (Expert 2 and 3) (Extended data- supplementary quotes, Table 4).

Despite requiring and utilising substantial resources, SSLE sustainability and cost-effectiveness may not be guaranteed. As reported by Expert 3, the facilitator had to convince a fishery community to participate in the exchange and had to compensate the community for the loss of value since they were not fishing during the visit exchange. Despite this, the exchange did not lead to the expected outcome - the community did not implement a conservation area. The benefit of the exchanges may not outweigh their cost.

Furthermore, interviewees highlighted that specific national South-South cooperation (SSC) budgets for SSLEs are often not provided by governments or international organizations. Exchanges are usually financed by organizations through their own funding or membership fees (Extended data- supplementary quotes- Table 4).

### Participant and donor reluctance

Prior to participating in SSLEs, participants and stakeholders alike often demonstrate little interest in SSLE programs. SSLE champions need to actively advocate for an exchange program and its added benefits:

Six years ago, we started talking about family planning integration and we started implementing it in Nepal, but many governments did not want to attend meetings around it. Once we have showed them [the results of the exchange], the SSLE generated their interest. Meetings and international meetings, that bring experience from other regions, are key. (Expert 5)

Experts encouraged the inclusion of the skeptical participants into the exchange. Once they are engaged, they would be able to convince other skeptics. Expert 3 illustrated the benefits of engaging a skeptical community in the exchange between fishing communities on re-conservation in Madagascar:

Selecting people who were skeptical about the SSLE sounds stupid. (…) These people will often be the first to oppose a management or conservation measure, so turning them into early advocates is enormously advantageous. (Expert 3)

Apart from stakeholders and participants, a barrier faced by organizations in promoting SSLEs is the limited national political commitment to and operationalization of SSLE as an alternative model. In addition to that, expert 9 highlighted the absence of national policy and strategic frameworks, as well as the absence of international coordination in South-South cooperation. New participants often do not have the motivation, time or willingness to take part in an exchange. Greater confidence and approaches from participants and governments may strengthen the SSLE’s organization and its operationalization.

### Absence of systematized and internationally recognized methodology

Nowadays, most organizations who gain experience in conducting SSLEs draft their own internal guide and tools on how to facilitate an exchange, as illustrated in the following quotes:

UNFPA has an internal guide document. The process can be divided into three distinct phases: 1) the consultation, 2) planning and implementation of activities; and 3) joint review of progress after one year. The Government commits to holding a consultation once a year. (Expert 1)
The process is not the same for every exchange, that is why we tended to give a flexible 10-steps framework in the guide. It does not depend on the objectives. Generally, you must have three clear objectives you want to achieve, you do an informal M&E, debriefing and follow-up when we finished. Over the years, we developed templates to help manage the exchange. (Expert 3)

The SSLE process is not systematized and is not documented. Participants do not reserve time to record their learnings and discuss them, even if this step is considered valuable and helps in growing and improving the participants skills and exchange results (Extended data- supplementary quotes, Table 5).

Regarding SSLE follow-ups (tracking results and reporting), the facilitator often conducts post evaluation, such as a follow-up survey or informal feedback from the participants, often in a unstandardised way (Extended data- supplementary quotes- Table 5). Regular follow-up meetings can facilitate and support the implementation of action plans, but several experts declared that these meetings are rarely organized due to budget constraints and the limited interest of participants.

During the SSLE, most experts developed an action plan or roadmap for implementing the know-how in their country/community and few of them monitored the process after the exchange. The Government of Indonesia, cooperating closely with partners from the Global South since 1955, has established a methodology and developed tools to annually report and evaluate all the activities during and after the SSTC programmes. Other organizations have developed their internal tools, as demonstrated in the following quotes:

During the exchange with the Philippines, we developed a five-year roadmap. Then, we evaluated [the progress] yearly because we held the steering committee meeting a year afterwards, where we evaluated last year’s program and developed next year’s program. (Expert 11)

Even if each organization developed its instruments and tools for implementing the gained knowledge, all the experts highlighted that monitoring and evaluating (M&E) is challenging and it is often omitted, as illustrated in the following quote:

The monitoring focused on the action plan, rather than monitoring progress post-broader M&E. (Expert 4)

Several agencies and institutions have developed their own methodology, frameworks, evaluation techniques and implementation strategies for SSLE that are often tailored to their specific contexts and are not widely recognized across different sectors.

## Discussion

This study sheds light on some strengths and weaknesses of South-South learning exchanges by examining the perspective of a range of experts from different disciplines. Empowerment of participants, positive peer-to-peer “mind change” and powerful knowhow are the main strengths of the SSLE approach. Resource heavy, reluctancy of participants and absence of a validated guide methodology emerged as main weaknesses of SSLE, which could impair the effectiveness of this approach. This study illustrated that SSLE is a promising and valuable tool to pass on knowledge and information from a grass-roots approach.

The SSLEs’ strengths derived from two main processes previously described in the management, educational and psychological literature: experiential learning and social learning.

SSLE provides participants with hands-on, personal experiences, that are key to experimental learning. As described in the Kolb’s experimental learning model ( Kolb, 2015) and its subsequent revision ( Morris, 2019), learning consists of a four-stage cycle that includes a concrete experience, a reflective observation on the experience, conceptualization, and an active experimentation of what you have learnt. “Learning by doing” has been highlighted as a funding concept of this approach by Morrison *et al.* ( Morris, 2019). Learners are immersed in this learning experience that contains context-specific information, and this “hands-on” process makes learners active and empowered. In fact, participants’ reflection on the acquired knowledge or experience and the internalization and application of the knowhow are the strengths of the South-South model. This approach leads to an understanding that is different from that acquired through research, observation, books, and lessons ( Borkman, 2007). Moreover, this “hands-on” process enhances ownership, empowerment, enthusiasm and leadership among participants.

During the exchange, participants are involved in the process of social learning ( Schusler *et al.*, 2003). The social learning theory postulated that “social behaviour is learned by observing and imitating the behaviour of others” and suggested that “behaviour change is more likely when modelling is provided by peers than non-peers” ( Bandura & RH, 1977). Interactions with peers who are successfully coping with their experiences are more likely to result in positive behaviour change and peers are more credible role models for others. Personal interactions created a common understanding and may encourage the continuous sharing of best practices after the exchange event ( Jenkins *et al.*, 2017). This study showed that participants from countries with similarities exchange knowledge and expertise in a convincing way, such as the exchange run among Indonesian and Chadian Muslim religious leaders on family planning. The peer model is a key strength of the SSLE.

South-South Learning Exchanges can be resource-intensive, and necessitates substantial commitment from participants and organizers. The major cost factors include expenses for human resources, travel, accommodation, and conference facilities - essential for conducting these exchanges. Adequate funding enables the increase of dedicated personnel and the enhancement of existing staff capabilities through training. More skilled personnel can help diversifying roles and responsibilities among participants, enhancing the quality of the process. SSLE financing sources are varied, influenced by the level of involvement and engagement of organizations and governments, and varied across different regions and countries, as well as thematic areas. Funding often comes from United Nations agencies (e.g. UNFPA, FAO, IFAD, PAHO), the World Bank, governments, non-governmental organizations (NGOs) and bilateral/multilateral aid entities. Some offices allocate specific portions of their core budget, while others rely on programmatic funds. Comprehensive data on the resources invested in and leveraged by SSLEs remains elusive, underscoring the need for more transparent and detailed reporting mechanisms. Although traditional learning exchanges are known to be costly and time-consuming ( Gardner *et al.*, 2017; Jenkins *et al.*, 2017), recent initiatives conducted by the co-authors show that SSLE can be successfully conducted online at reduced costs ( Kabra *et al.*, 2022). However, the participants reported that that virtual formats lack the impact of in-person interactions, which offer direct, hands-on learning experiences. Achieving the desired outcomes of SSLEs thus requires significant investment, underscoring the importance of sustained national and international commitment, as well as partnerships with international bodies and the private sector for funding.

The reluctance of participants and donors to engage with SSLEs can be attributed to several challenges inherent in the implementation of SSLE initiatives. These challenges include a lack of standardized approaches, frameworks, and evaluation methods, as well as limited evidence demonstrating the impact of these exchanges ( Olu *et al.*, 2017; UNFPA Evaluation Office, 2020). Frequently, SSLE programs are characterized by their small scale, informal nature, or integration within broader cooperation programs ( WHO & World Health Organization, 2014), with the documentation and dissemination of outcomes often being overlooked. Our analysis indicates that diverse approaches and frameworks are implemented across different agencies and organizations, leading to monitoring, evaluation, and follow-up processes often seen as inadequate and variegated across different sectors. This inadequacy is underscored in the “Formative evaluation of UNFPA approach to South-South and triangular cooperation” ( UNFPA Evaluation Office, 2020) and by other scholars, such as Jenkins *et al.* and Thompson *et al.* ( Jenkins *et al.*, 2017; Thompson *et al.*, 2017). However, exceptions illustrate the potential for success through rigorous methodologies. For instance, the Korean Development Institute and the World Bank Institute have showcased the effectiveness of a results-focused approach in assessing knowledge exchange programs through three detailed case studies ( Chun & Kim, 2011). Similarly, collaborative efforts between Nepal and Sri Lanka, under WHO’s facilitation, have led to the development of a monitoring tracker and framework to effectively implement action plans during SSLE ( Kabra *et al.*, 2022). These examples demonstrate that employing a thorough methodology, including a strong emphasis on the preparation phase (prioritization of the learning objectives) and on the post-exchange implementation (i.e. meticulous documentation and monitoring & evaluation), can significantly enhance the assessment and demonstration of SSLE impacts. Such robust methods increase the credibility and visibility of SSLE initiatives among participants, governments, and funders, ultimately leading to better access to and availability of learning resources through SSLE. Moreover, the development of a standardized and cross-sector methodology, frameworks and evaluation system allows to better measure and assess the contribution that a country or institution make in international development, as well as to reflect on pitfalls encountered during the SSLE. Countries from the Global South should lead the development of a methodology and ensure that these tools are tailored to their specific contexts and needs ( Besharati & MacFeely, 2019). However, Waisbich LT highlights that the debate on measuring South-South Cooperation (SSC) unveils intricate and unresolved discussions between the Global North and South (or the South and the rising powers, such as Brazil, China, India) on "power, status, and responsibilities" within international development—a complexity that extends beyond the scope of this paper ( Waisbich, 2022). While regular monitoring and evaluation are critical for enhancing SSLEs, the underlying tensions and disagreements between the North and South persist, suggesting these challenges may continue to influence the dialogue on SSC.

We note several limitations of this study. Firstly, participants were identified through reports and documents found in our scoping review ( Allagh *et al.*, 2023). Although we included a variety of publications, we might have overlooked individuals involved in SSLEs, potentially introducing a selection bias into our research. Additionally, participants were selected from available SSLE organizers or participants whom the authors could contact, leading to challenges in recruiting interviewees since we were solely able to contact them by email. Then, as most of interviewees were SSLE organizers or facilitators, we were unable to capture insights from the peer teams and stakeholders’ into the current study. This may have potential implications that should be emphasised in future studies. Conducting interviews exclusively in English may have been a potential limitation for our research, even if it's essential to note that all the participants were able to engage effectively. Future research should consider language diversity. Finally, recruitment was done by the authors of this study who work at the SRHR department, and several interviewees were SRHR experts; there may be some specific bias in recruitment.

To our knowledge, this is the first manuscript exploring the perspectives of a sample of experts on SSLE from different disciplines. The originality of this study lies in the collection of various personal and professional SSLE experiences: we sampled across various professional levels (government representatives, NGOs, communities) and captured the far-ranging capacities of SSLE application (from fishery management to family planning). An additional strength of this study stems in part from the interdisciplinary team. Co-authors of this study are from different disciplines such as healthcare management, public health, human reproduction and family planning.

This manuscript has highlighted the need for reaching a consensus on guidelines, framework and an evaluation system to conduct and assess the impact of SSLE. Despite several initiatives being conducted all over the world, they are not comparable in terms of adopted guidelines, documentation, frameworks and evaluation measures. By focusing research efforts on these areas (i.e. Delphi technique, consensus conferences), it will be possible to implement standardized approaches and metrics for evaluating both the short-term and long-term impacts of SSLEs, as well as their cost benefit. This will not only enhance our understanding of SSLEs but also ensure their benefits are maximized and efficiently realized. The initial version of the five-step methodology developed by the WHO mark a significant stride toward a unified approach, highlighting a strong focus on the defining the learning objectives and emphasizing the post-exchange implementation phase. Drawing on the insights from this paper and our accumulated experience in the field, we have compiled a set of key recommendations to guide and enhance future SSLEs.

**Box: Key Recommendations**

**For facilitators:**

*Preparation phase:*

Before initiating an SSLE, it is crucial to invest significant effort in the preparation phase, specifically in the prioritization and definition of the learning objectives. This step is foundational for achieving the expected results and outcomes. Teams should conduct a comprehensive needs assessment to identify key areas where knowledge sharing could yield the most significant impact. Moreover, involving stakeholders and experts in developing a structured framework can help ensure that the learning question is relevant, focused, and capable of guiding the exchange towards meaningful outcomes.

Select and empower influential champions who can lead the exchange, driving meaningful change and advocating for it.

Match teams with similar socio-economic backgrounds to create an environment conducive to positive mindset changes, thereby facilitating the effective application of learned insights.

Assign specific responsibilities within the team or designate dedicated individuals to oversee tasks, including the documentation and M&E process.

*Exchange*:

Establish a space that encourages participants to engage in constructive dialogue, promoting empathy, diversity, and a sense of solidarity. This approach will empower participants by fostering connections and understanding, ensuring a more impactful exchange experience.

*Post-exchange phase:*

After the completion of a study tours, it is essential to focus on post-exchange activities, including the implementation of learning, follow-up with participants, and the documentation of outcomes and insights.

Establish a protocol for capturing the lessons learned and the impacts observed, incorporating both quantitative and qualitative data to strengthen this stage. This documentation should be made accessible to a wider audience, as a valuable resource for future SSLEs.

Establishing a regular follow-up mechanism can help in assessing the long-term effectiveness of the exchange and facilitating continuous learning and improvement. For example, organizing follow-up meetings and advocating for the dissemination of acquired knowledge back in their respective countries or communities support the implementation of learnings, emphasizing their importance in addressing changes and maintaining participant enthusiasm.

**At the international level:**

Working towards a more unified consensus on the definition of SSLE and a systematic methodology is key for all actors involved in SSLE.

Establishing a community of practice on SSLEs and developing a platform dedicated to gather SSLE experiences could facilitate knowledge sharing and promote transparency.

For governments and institutions from the Global South, prioritize the development of policies on SSLE, underpinned by a strategic framework grounded in evidence-based practices.

## Conclusion

This study has shed light on the multifaceted nature of South-South Learning Exchanges (SSLE), uncovering both strengths, such as participant empowerment, positive peer-to-peer mindset shifts, and the transfer of hands-on learning, and weaknesses, including the resource-intensive nature of these exchanges, participant reluctance, and the lack of a validated guiding methodology. The strengths of SSLEs are anchored in the theories of experiential and social learning, highlighting SSLE's potential to create an environment that significantly enhances knowledge exchange among participants. Despite these strengths, the study highlights the challenges SSLE initiatives face, particularly the limited funds and commitment to support them, stemming from limited evidence of impact, disparate approaches, and the absence of standardized guidelines, and evaluation practices., The development of a comprehensive set of guidelines, frameworks, and evaluation systems will not only enhance our understanding of SSLEs, but also extend their benefits and ensure their effective implementation.

## Data Availability

In order to protect the privacy of the participants, the full data containing identifiable information has not been made publicly available. However, researchers in a related field can request additional details about the interviewees. To obtain the data, interested researchers should send an email to isotta.triulzi@santannapisa.it with the subject line 'Strengths and weaknesses of SSLE' and explain their reason for needing the data. They must also confirm that the data will not be made public or misused, and that the sharing is documented. Repository: *Experts’ perspectives on strengths and weaknesses of the South- South Learning Exchange: a qualitative analysis*. DOI: 10.6084/m9.figshare.22742180 This project contains the following extended data: Interview Guide Supplementary Quotes Data are available under the terms of the Creative Commons Zero "No rights reserved" data waiver (CC0 1.0 Public domain dedication). Repository: Consolidated Criteria for Reporting Qualitative Research (COREQ) check list for *Experts’ perspectives on strengths and weaknesses of the South- South Learning Exchange: a qualitative analysis*. DOI: 10.6084/m9.figshare.23045015 Data are available under the terms of the Creative Commons Zero "No rights reserved" data waiver (CC0 1.0 Public domain dedication).
