# Supplementary material for: Strengths and weaknesses of the South-South Learning Exchange: a qualitative analysis of experts’ perspectives
Source: Gates Open Res. 2024 Mar 27;7:116. Originally published 2023 Aug 18. [Version 2] doi: 10.12688/gatesopenres.14699.2 (PMC11259588; doi:10.12688/gatesopenres.14699.2)
Supplement: Peer review history

# Reviewer report: Reviewer response for version 2

**DOI:** 10.21956/gatesopenres.16705.r36912

**Published:** 2024-07-19

**Authors:** Nikolas Fajar Wuryaningrat

**Affiliations:** 1 Manado State University, Sulawesi Utara, Indonesia; 2 Borneo Tarakan University (Ringgold ID: 175494), Tarakan, East Kalimantan, Indonesia

It is recommended that the novelty and urgency of this research be reinforced, as the information currently submitted is not sufficiently clear about the problems and urgency.

 Once the information pertaining to the problem, novelty and urgency has been enhanced, it is essential that the research results submitted are capable of addressing the problem or urgency of the research.

Is the work clearly and accurately presented and does it cite the current literature?

Yes

If applicable, is the statistical analysis and its interpretation appropriate?

Not applicable

Are all the source data underlying the results available to ensure full reproducibility?

No source data required

Is the study design appropriate and is the work technically sound?

Yes

Are the conclusions drawn adequately supported by the results?

Partly

Are sufficient details of methods and analysis provided to allow replication by others?

Yes

Reviewer Expertise:

Knowledge Management, Innovation and Entrepreneurship

I confirm that I have read this submission and believe that I have an appropriate level of expertise to confirm that it is of an acceptable scientific standard, however I have significant reservations, as outlined above.

# Reviewer report: Reviewer response for version 2

**DOI:** 10.21956/gatesopenres.16705.r37261

**Published:** 2024-07-19

**Authors:** Hanna Kontio

**Affiliations:** 1 University of Helsinki, Helsinki, Finland

The article presents findings from an interview study on South-South Learning Exchanges (SSLE) in the field of Family Planning. The interviewees include experts working in international organisations, governmental and non-governmental organisations in various continents, with emphasis on experts located in the countries of the 'global South'. Connecting the aims to the WHO guidelines on SSLE, the analysis aims at identifying both strengths and weaknesses in the current SSLE practices. The results indicate both of these in a balanced manner. The analysed experiences point to the importance of careful planning and preparation of the exchanges, sensitivity to local context and inter-personal dynamics and a careful follow up as elements to meaningful and beneficial SSLE practices. The qualitative insights can hopefully inform development of practice and guidelines for SSLE. The study is adequately reported and its limitations are transparently discussed. The paper provides a good contribution to practitioners of SSLE and is of interest to the academic community studying global exchanges.

Is the work clearly and accurately presented and does it cite the current literature?

Yes

If applicable, is the statistical analysis and its interpretation appropriate?

Not applicable

Are all the source data underlying the results available to ensure full reproducibility?

Partly

Is the study design appropriate and is the work technically sound?

Yes

Are the conclusions drawn adequately supported by the results?

Yes

Are sufficient details of methods and analysis provided to allow replication by others?

Yes

Reviewer Expertise:

international partnerships, intercultural learning, collaborative learning, research for development

I confirm that I have read this submission and believe that I have an appropriate level of expertise to confirm that it is of an acceptable scientific standard.

# Reviewer report: Reviewer response for version 2

**DOI:** 10.21956/gatesopenres.16705.r36273

**Published:** 2024-03-29

**Authors:** Siddhartha Paul Tiwari

**Affiliations:** 1 Google Asia Pacific Pte Ltd, Singapore, Singapore

No new comments, well done overall

Is the work clearly and accurately presented and does it cite the current literature?

Yes

If applicable, is the statistical analysis and its interpretation appropriate?

Yes

Are all the source data underlying the results available to ensure full reproducibility?

Yes

Is the study design appropriate and is the work technically sound?

Yes

Are the conclusions drawn adequately supported by the results?

Yes

Are sufficient details of methods and analysis provided to allow replication by others?

Yes

Reviewer Expertise:

Knowledge sharing, Learning exchange, peer-to-peer learning

I confirm that I have read this submission and believe that I have an appropriate level of expertise to confirm that it is of an acceptable scientific standard.

# Reviewer report: Reviewer response for version 1

**DOI:** 10.21956/gatesopenres.16024.r35273

**Published:** 2023-10-31

**Authors:** Siddhartha Paul Tiwari

**Affiliations:** 1 Google Asia Pacific Pte Ltd, Singapore, Singapore

**Brief description of the article and its relevance**

 A 360 degree understanding of SSLE is demonstrated in the article as a result of thorough and time-consuming research. As a result, the article is well-written and informative. As a peer-driven and interactive learning process, South-South learning exchange (SSLE) aims to foster positive change by engaging participants in a peer-driven knowledge-sharing process. Despite the fact that organizations across the world have offered more opportunities for SSLE, the methods and support systems for this type of learning have not been extensively researched or documented in scientific papers. This is a thrust area for future research. This study contains a considerable amount of research on the strengths, weaknesses, and mechanisms of SSLE, as well as expert opinions. Participants' empowerment, positive peer-to-peer "mind change," and the power of hands-on learning were all observed as strengths in the successful implementation of SSLE, as stated in experts' accounts. The originality of this study is apparent, as it is a niche topic.

**Major Observations**

  

1. The study gains a higher degree of credibility when authentic and independent sources are referred to, such as the WHO, UNDP, and The World Bank, which lend credence to the study. The article is very well organized. It has logical sections that build on each other, making it easy for the reader to follow and understand. Your inclusion of key literature not only validates the points mentioned in the article, but also places the study in a broader academic context.
2. Using the example of SSLE between Nepalese and Sri Lankan ministries of health, it is possible to obtain a practical perspective on the topic and concepts in terms of tangible outcomes. It may be necessary to examine the experiences of countries or organizations that did not find SSLE beneficial or faced roadblocks when implementing SSLE in order to provide a balanced perspective.
3. It is mentioned that there were three authors who supervised the coding of the data; however, the methodology could be strengthened by clarifying how disagreements were resolved or if any challenges were encountered in maintaining consistency. If there were no disagreements or challenges during the process, a mere mention of that may also help and strengthen the methodology.
4. As an indicator of the quality of the output, the average length of the interviews may be useful. Even though a range of 30 to 60 minutes is provided, the average may provide a more accurate representation of how long a typical interview session took.

**Minor Observations**

1. For the conclusion, it may be possible to improve it by summarizing the study slightly. A succinct summarization of the key findings and their implications might be helpful toward the end of the article, in order to conclude.
2. The conclusion points out that this approach requires national funding to improve its accessibility and availability. However, the conclusion mentioned in the ‘abstract’ mentions both national and international funding. It may be worthwhile to consider or at least mention other potential mechanisms of support, such as international partnerships or private sector collaborations, that may also help SSLE. A more detailed explanation of why funds are so important and how they can uniquely strengthen SSLE would provide greater depth to the conclusion.
3. While the article discusses the need for further research on SSLE's long-term impact and sustainability. The recommendations for future research may need to be made more explicit in order to ensure their success. For instance, a) Is there a specific methodology they should follow in order to accomplish this? b) Are there any knowledge gaps they need to fill? c) If so, what should they do?

Is the work clearly and accurately presented and does it cite the current literature?

Yes

If applicable, is the statistical analysis and its interpretation appropriate?

Yes

Are all the source data underlying the results available to ensure full reproducibility?

Yes

Is the study design appropriate and is the work technically sound?

Yes

Are the conclusions drawn adequately supported by the results?

Yes

Are sufficient details of methods and analysis provided to allow replication by others?

Yes

Reviewer Expertise:

Knowledge sharing, Learning exchange, peer-to-peer learning

I confirm that I have read this submission and believe that I have an appropriate level of expertise to confirm that it is of an acceptable scientific standard.

# Response

**Published:** 2024-03-11

**Authors:** Isotta Triulzi

**Affiliations:** Scuola Superiore Sant'Anna di Studi Universitari e di Perfezionamento, Italy

Dear Siddhartha Paul Tiwari

 Thank you very much for your positive and encouraging feedback. The queries and the suggestions you also provided were beneficial. Below is our response to your comments and the requests you make.

**Major Observations**

***The study gains a higher degree of credibility when authentic and independent sources are referred to, such as the WHO, UNDP, and The World Bank, which lend credence to the study. The article is very well organized. It has logical sections that build on each other, making it easy for the reader to follow and understand. Your inclusion of key literature not only validates the points mentioned in the article, but also places the study in a broader academic context.***

**Response: **Thank you for your insightful feedback. We are pleased to hear that you found the manuscript well-organized and easy to navigate, and that the inclusion of reputable sources alongside key literature, has enhanced the credibility and academic context of our study. We are grateful for your appreciation.

***Using the example of SSLE between Nepalese and Sri Lankan ministries of health, it is possible to obtain a practical perspective on the topic and concepts in terms of tangible outcomes. It may be necessary to examine the experiences of countries or organizations that did not find SSLE beneficial or faced roadblocks when implementing SSLE in order to provide a balanced perspective.***

**Response: **We have explored a range of exchanges through our interviews, and some participants shared that they did face various challenges. These insights are detailed in the results section of the manuscript, providing a comprehensive view of the diverse experiences associated with SSLE implementations.

**It is mentioned that there were three authors who supervised the coding of the data; however, the methodology could be strengthened by clarifying how disagreements were resolved or if any challenges were encountered in maintaining consistency. If there were no disagreements or challenges during the process, a mere mention of that may also help strengthen the methodology.**

**Response: **Thank you for this comment. We have reviewed the methodology accordingly. Please check it.

***As an indicator of the quality of the output, the average length of the interviews may be useful. Even though a range of 30 to 60 minutes is provided, the average may provide a more accurate representation of how long a typical interview session took.***

**Response: **We have incorporated this information within the revised manuscripts. The average length of the interview was 50 minutes.

**Minor Observations**

***For the conclusion, it may be possible to improve it by summarizing the study slightly. A succinct summarization of the key findings and their implications might be helpful toward the end of the article, in order to conclude.***

**Response: **We have rewritten the conclusion summarizing the key findings and their implications, as per your suggestions.

***The conclusion points out that this approach requires national funding to improve its accessibility and availability. However, the conclusion mentioned in the ‘abstract’ mentions both national and international funding. It may be worthwhile to consider or at least mention other potential mechanisms of support, such as international partnerships or private sector collaborations, that may also help SSLE. A more detailed explanation of why funds are so important and how they can uniquely strengthen SSLE would provide greater depth to the conclusion.***

**Response: **Thank you for your constructive feedback on the significance of funding for SSLE. We appreciate this chance to clarify and deepen our discussion on this topic. In response, we have updated the discussion section to offer a more detailed examination of the various types of funding available and their pivotal role in strengthening SSLE initiatives. This includes an exploration of national and international funding sources, as well as the potential for support through international partnerships and private-sector collaborations. Additionally, we have revised the abstract to ensure consistency and clarity.

***While the article discusses the need for further research on SSLE's long-term impact and sustainability. The recommendations for future research may need to be made more explicit in order to ensure their success. For instance, a) Is there a specific methodology they should follow in order to accomplish this? b) Are there any knowledge gaps they need to fill? c) If so, what should they do?***

**Response: **Thank you for this comment. We have enriched the paragraph at the end of the discussion, and we have highlighted the need for more research on developing consistent guidelines, frameworks and evaluation systems in SSLE.

# Reviewer report: Reviewer response for version 1

**DOI:** 10.21956/gatesopenres.16024.r34852

**Published:** 2023-09-29

**Authors:** Jane Hirst

**Affiliations:** 1 Nuffield Department of Women's & Reproductive Health, University of Oxford, Oxford, England, UK

This is an interesting article with important learnings around south-south learning exchange.

- It would be useful to state what levels the SSLE explored in this paper relate to: e.g. government to government; subnational level to subnational; NGO to NGO or community to community, or combinations? Could this be a factors in success or failure of the program?
- One of the comments refers to power imbalance being a limiting factor in the success of the SSLE (the Mexico-Malagasi fishing example). Could the authors comments on the level of engagement that the case studies they explore relate to and whether that had any bearing on the perceived or actual impact of the exchange.
- The authors come from a health background, specifically in contraception. Are there are factors which make SSLE in this sector any more of less likely to be successful compared to the other sectors?
- It would be useful for someone not in this field to define the different between a participant and a stakeholder in SSLE.
- The lack of an agreed framework for SSLE is listed as a limitation, yet it appeared several of the larger multilaterals or NGOs in this paper have their own frameworks, including WHO. Are these frameworks not known or not used? It would be useful in the discussion to consider what is needed to move beyond this situation. Is the situation limited to certain sectors, or is it common across all?
- The participants involved in the interviews were mostly facilitators of the process. Are there any similar studies looking at the perspectives of the peer teams or stakeholders? The views of the facilitators are valuable, however only represent one dimension to the experience.
- In table 1, it would be helpful to list the years’ experience or number of SSLE each participant had been involved in to give an idea or how “expert” the Experts actually were
-  In the introduction it would be useful to give the headline finding of your scoping review in this aera, how many programs did you identify and the key message
- As the objective is stated that this is the final step in the scoping review process as key informant interviews with stakeholders, please justify the inclusion of interviewees not in the FP space (i.e. fisheries)
- Were the stakeholders who attended the 2021 WHO consultation the same as the interviewees in this paper? It is a little unclear which stakeholder you are referring to.
- What were the limitations selecting only authors identified in your scoping review? Presumably most SSLE don’t result in a peer-reviewed publication so could this have introduced bias? How did you ensure geographic representation in this exercise? Please state in the description of the participants how many were LMIC (i.e. “South”) based.
- In the data collection section, more should be included to indicate reflexivity of the lead researchers and her own personal positionality and experience of SSLE, including whether she was commissioned/paid to do this work.
-  After the interview guide piloting were any changes made? Did the interviewer adapt the interview guide based on the findings of previous interviews?
- Could only conducting the interviews in English been a limitation for this process?
- From the case studies described, I was left a bit unclear about what comprises a SSLE. Some seemed to have study tours, others a facilitated session. Is there a clear definition of what constitutes a SSLE and how long it needs to be at a minimum? Are all SSLE bilateral, or are some trilateral, multilateral, regional etc. Is a regional conference a SSLE for example or is what distinguishes it a predefined implementation agenda?
- COVID 19 outbreak started in March 2020 globally, not March 2019
- Zoom, WhatsApp and Microsoft Teams are all brand names and should be capitalised and if appropriate a TM or company name indicated
- Cost is always a limitation. For the reader less familiar with this space, it would be useful to describe the range of sources and scale of funding the experts used for these activities.
- What would help really bring this paper together would be a box of key recommendations for those conducting and planning SSLE based on your findings.

Is the work clearly and accurately presented and does it cite the current literature?

Yes

If applicable, is the statistical analysis and its interpretation appropriate?

Not applicable

Are all the source data underlying the results available to ensure full reproducibility?

Yes

Is the study design appropriate and is the work technically sound?

Yes

Are the conclusions drawn adequately supported by the results?

Yes

Are sufficient details of methods and analysis provided to allow replication by others?

Partly

Reviewer Expertise:

Global women's health, health systems

I confirm that I have read this submission and believe that I have an appropriate level of expertise to confirm that it is of an acceptable scientific standard, however I have significant reservations, as outlined above.

# Response

**Published:** 2024-03-11

**Authors:** Isotta Triulzi

**Affiliations:** Scuola Superiore Sant'Anna di Studi Universitari e di Perfezionamento, Italy

Dear Prof. Jane Hirst,

 We greatly appreciate the time and effort you've dedicated to providing thoughtful comments and suggestions on our study. We have dedicated our efforts to address your concerns and hope that our revisions reflect the high standards you uphold.

 We look forward to any further suggestions you may have that can enhance our study.

 Best wishes

**1. *It would be useful to state what levels the SSLE explored in this paper relate to: e.g. government to government; subnational level to subnational; NGO to NGO or community to community, or combinations? Could this be a factor in success or failure of the program?***

**Response: **Thank you for this comment.  The exchange was a combination of staff from different levels, government, Sub national level staff, academia, NGO . We have enriched *Table 1.Characteristic of study participants* with additional information and we have included further information on the type of the exchanges and main participants that indicate the level of each SSLE.

**2. *One of the comments refers to power imbalance being a limiting factor in the success of the SSLE (the Mexico-Malagasi fishing example). Could the authors comments on the level of engagement that the case studies they explore relate to and whether that had any bearing on the perceived or actual impact of the exchange.***

**Response: **The level of participant engagement in the SSLEs plays a crucial role in facilitating the exchange of information and implementing the learnings.  In most SSLE s ministry of staff has been active participant and this actually worked as a motivating factor. As highlighted in our manuscript, empowering participants emerges as a key strength of these exchanges. Participants who showed high levels of engagement (referred to as champions) are more motivated to have advocate for change upon returning to their team.

**3. *The authors come from a health background, specifically in contraception. Are there factors which make SSLE in this sector any more of less likely to be successful compared to the other sectors?***

**Response: **Thank you for your comment. Considering the factors that may influence the success of the South-South Learning Exchange (SSLE) in the field of contraception, we recognised the challenges posed by cultural and religious factors. Contraception is closely intertwined with personal beliefs and practices, often involving couples and communities. Navigating these sensitive and complex dynamics is essential for effective knowledge exchange in this sector. While we recognize that other sectors such as agriculture and fishing have their own challenges, we believe that addressing the specific cultural and interpersonal aspects of contraceptive practices requires a standardised yet tailored and culturally competent approach within the SSLE. Thank you for highlighting this important aspect.

**4. *It would be useful for someone not in this field to define the difference between a participant and a stakeholder in SSLE.***

**Response: **Thank you for raising this important point. In the context of the South-South Learning Exchange (SSLE), defining the difference between a participant and a stakeholder is crucial for clarity. A participant typically refers to an individual or group directly engaged in the learning exchange activities, such as attending visit tours, study tours, virtual meetings within the SSLE framework. On the other hand, a stakeholder encompasses a broader range of individuals or organizations who support or have an interest in, or may be affected by, the outcomes of the SSLE. This could include policymakers, government agencies, non-governmental organizations, community representatives, and other relevant entities whose involvement and influence extend beyond direct participation. We have clarified on these distinctions in our revised manuscript (introduction) to ensure a clear understanding for readers beyond the field. Thank you for highlighting the need for this clarification.

**5. *The lack of an agreed framework for SSLE is listed as a limitation, yet it appeared several of the larger multilaterals or NGOs in this paper have their own frameworks, including WHO. Are these frameworks not known or not used? It would be useful in the discussion to consider what is needed to move beyond this situation. Is the situation limited to certain sectors, or is it common across all?***

**Response: **Several agencies and non-governmental organizations, the World Health Organization (WHO) included, have embarked on SSLEs employing a variety of methodologies and frameworks. Drawing on this diversity of approaches, our research introduces a five-step methodology specifically designed to enhance the effectiveness and impact of SSLEs.

 Our methodology places a strong emphasis on the preparation phase, particularly on the prioritization of the learning question, which we believe is crucial for setting the stage for a successful exchange. This initial step ensures that all participants are aligned and focused on the objectives of the exchange. Furthermore, we give considerable attention to the phases following the exchange. This includes post-exchange implementation, follow-up, and comprehensive documentation of the outcomes and lessons learned. Our approach is designed not only to maximize the immediate benefits of the exchange for the participating entities but also to provide a valuable resource for others in the field.

**6. *The participants involved in the interviews were mostly facilitators of the process. Are there any similar studies looking at the perspectives of the peer teams or stakeholders? The views of the facilitators are valuable, however only represent one dimension to the experience.***

**Response: **Thank you for this comment. In response to your inquiry about similar studies that explore the perspectives of peer teams or stakeholders, we identified several pieces of literature, including a study on SSLE in marine conservation by Gardner et al. (2017), where the views of peer teams were examined through interviews. We recognize the importance of capturing a multi-dimensional perspective and have acknowledged this limitation in the discussion section of our revised manuscript.

**7. *In table 1, it would be helpful to list the years’ experience or number of SSLE each participant had been involved in to give an idea or how “expert” the Experts actually were***

**Response: **Thank you for the suggestion. We appreciate your feedback on incorporating details about the years of experience or the number of SSLE each participant has been involved in, to shed light on their expertise level. We have enriched Table 1 with additional information on the SSLE initiatives implemented by our sample of experts, including their years of experience (< 5 years; >5 years). Even if we did not inquire about the exact number of years, all the interviewees were senior professionals, demonstrating an extensive experience in this sectors.

**8. *In the introduction it would be useful to give the headline finding of your scoping review in this aera, how many programs did you identify and the key message***

**Response: **We have reviewed the introduction accordingly.

**9. *As the objective is stated that this is the final step in the scoping review process as key informant interviews with stakeholders, please justify the inclusion of interviewees not in the FP space (i.e. fisheries)***

**Response: **We have expanded our sample deliberately to encompass a wider scope, focusing on the SSLE process, methodology, and approach. These aspects extend beyond the confines of the FP space, and by including stakeholders from diverse fields, we aim to gain insights that contribute to a comprehensive understanding. Our goal is to explore best practices, and potential lessons that may not be confined to a specific sector. We thought that this approach allows us to enrich our scoping review with a more inclusive and holistic perspective.

**10. *Were the stakeholders who attended the 2021 WHO consultation the same as the interviewees in this paper? It is a little unclear which stakeholder you are referring to.***

**Response: **Yes, all of them were invited at the 2021 WHO consultation, but some of them attended it.

**11. *What were the limitations selecting only authors identified in your scoping review? Presumably most SSLE don’t result in a peer-reviewed publication so could this have introduced bias?***

**Response: **Our scoping review research strategy adopted a broad approach, and we included various type of publications identified via databases, registries and websites, and organizations. This type of research enables us to include participants from various fields, even if their experiences did not culminate in a peer-reviewed publication. However, we may overlooked individuals involved in SSLEs, potentially introducing a selection bias into our research, introducing a bias. We have clarified it in the text (section Methodology, Interviews ) and add it in the limitation section.

**12. *How did you ensure geographic representation in this exercise? Please state in the description of the participants how many were LMIC (i.e. “South”) based.***

**Response: **We have added a column in the table 1, country of birth, and a sentence in the results (Expert characteristics).

**13. *In the data collection section, more should be included to indicate reflexivity of the lead researchers and her own personal positionality and experience of SSLE, including whether she was commissioned/paid to do this work.***

**Response: **We have reviewed the text accordingly. This study was not commissioned and paid by the WHO.

**14. *After the interview guide piloting were any changes made? Did the interviewer adapt the interview guide based on the findings of previous interviews?***

**Response: **In response to your comment, only a few changes were made to the interview guide following the initial couple of interviews. These adjustments have been duly noted and incorporated into the manuscript, specifically in the Methodology section under data collection

**15. *Could only conducting the interviews in English been a limitation for this process?***

**Response: **While conducting interviews exclusively in English may have been a potential limitation for our research, it's essential to note that the vast majority of participants were able to engage effectively. However, we did encounter challenges with this sample of experts. We have mentioned this in the Discussion section (limitations).

**16. *From the case studies described, I was left a bit unclear about what comprises a SSLE. Some seemed to have study tours, others a facilitated session. Is there a clear definition of what constitutes a SSLE and how long it needs to be at a minimum? Are all SSLE bilateral, or are some trilateral, multilateral, regional etc. Is a regional conference a SSLE for example or is what distinguishes it a predefined implementation agenda?***

**Response: **Thank you for your thoughtful questions about SSLEs. Our scoping review (Allagh KP, 2023) captures four types of approaches including study tours (reciprocal, non-reciprocal), virtual exchange, expert visits, and mixed method exchange (study tour and expert visits; virtual reciprocal exchange), and it has emphasized that SSLEs can be bilateral, trilateral, or multilateral. SSLEs can manifest in bilateral, trilateral, multilateral, or even regional formats. The duration of SSLEs is not universally predefined, allowing for flexibility to tailor the exchanges according to the specific requirements and objectives of the participants. This information has been included in the revised manuscript.

**17. *COVID 19 outbreak started in March 2020 globally, not March 2019***

**Response: **This is corrected.

**18. *Zoom, WhatsApp and Microsoft Teams are all brand names and should be capitalised and if appropriate a TM or company name indicated***

**Response: **We have modified the text accordingly.

**19. *Cost is always a limitation. For the reader less familiar with this space, it would be useful to describe the range of sources and scale of funding the experts used for these activities.***

**Response: **Thank you for your suggestion. In response, we have expanded the discussion section to detail the variety of funding sources utilized for these exchanges. However, we must note that the specific amounts of funding employed by each agencies/experts were not requested during the interview. This information has been included in the discussion.

**20. *What would help really bring this paper together would be a box of key recommendations for those conducting and planning SSLE based on your findings.***

**Response: **We have added a box of key recommendations at the end of the results, as per your suggestions.
